# Real-time monitoring of molten zinc splatter using machine learning-based computer vision

**DOI:** 10.1007/s10845-024-02418-y

**Published:** 2024-05-22

**Authors:** Callum O’Donovan, Cinzia Giannetti, Cameron Pleydell-Pearce

**Affiliations:** Faculty of Science and Engineering, Swansea University, Fabian Way, Swansea, SA1 8EN Wales

**Keywords:** Galvanisation, Steel manufacturing, Computer vision, Deep learning

## Abstract

During steel galvanisation, immersing steel strip into molten zinc forms a protective coating. Uniform coating thickness is crucial for quality and is achieved using air knives which wipe off excess zinc. At high strip speeds, zinc splatters onto equipment, causing defects and downtime. Parameters such as knife positioning and air pressure influence splatter severity and can be optimised to reduce it. Therefore, this paper proposes a system that converges computer vision and manufacturing whilst addressing some challenges of real-time monitoring in harsh industrial environments, such as the extreme heat, metallic dust, dynamic machinery and high-speed processing at the galvanising site. The approach is primarily comprised of the Counting (CNT) background subtraction algorithm and YOLOv5, which together ensure robustness to noise produced by heat distortion and dust, as well as adaptability to the highly dynamic environment. The YOLOv5 element achieved precision, recall and mean average precision (mAP) values of 1. When validated against operator judgement using mean average error (MAE), interquartile range, median and scatter plot analysis, it was found that there was more discrepancy between the two operators than the operators and the model.This research also strategises the deployment process for integration into the galvanising line. The model proposed allows real-time monitoring and quantification of splatter severity which provides valuable insights into root-cause analysis, process optimisation and maintenance strategies. This research contributes to the digital transformation of manufacturing and whilst solving a current problem, also plants the seed for many other novel applications.

## Introduction

**Fig. 1:**
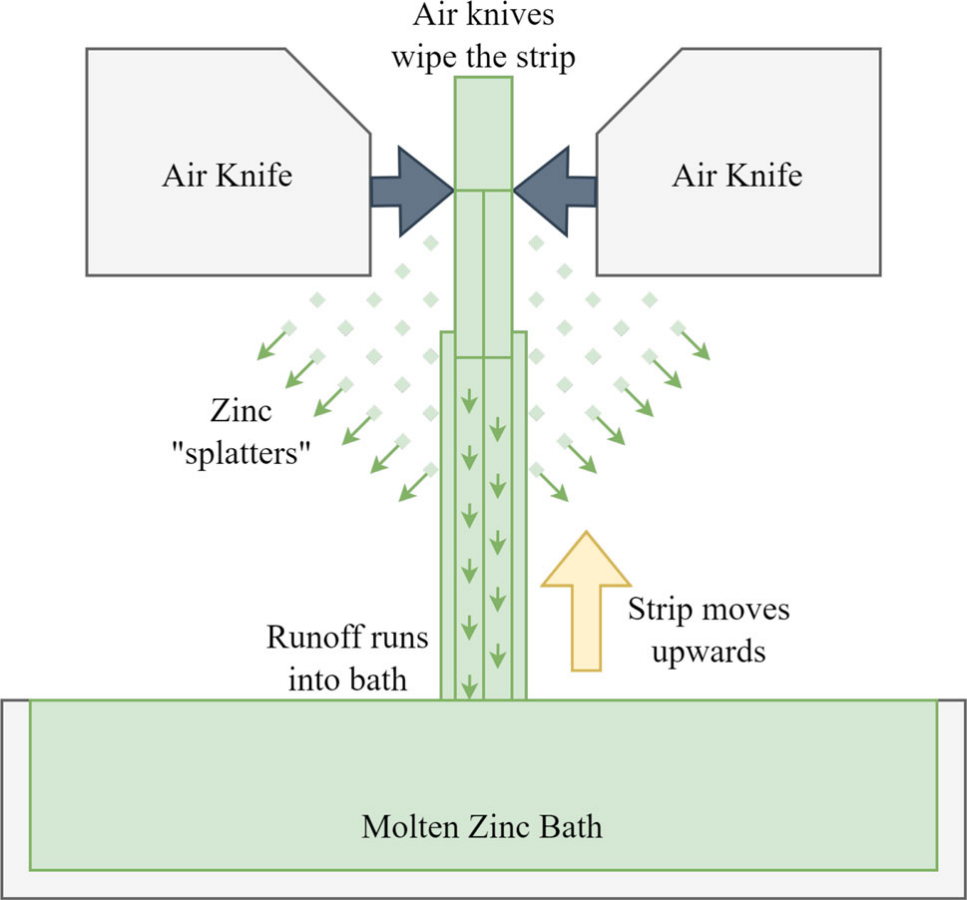
A schematic representing the part of the galvanising process where zinc splatter occurs

Immersion of preheated steel strip into a molten zinc bath is a critical step that occurs during galvanising. During this step, a zinc-iron alloy forms on the steel surface, increasing corrosion resistance dramatically. This process is carefully controlled to ensure constant uniform thickness, which is crucial to ensure proper protection from corrosion, to maintain a good aesthetic and structural integrity and also to ensure the output is predictable. The schematic shown in Fig. 1 represents this stage of the galvanising process. After the substrate is immersed in the zinc bath, which is maintained at around $450^\circ$ C, the coating weight thickness is controlled by a pair of air knives that wipe the strip as it leaves the zinc bath. The excess zinc (runoff) normally flows smoothly down the strip back into the zinc bath, however, at high strip speeds (typically meaning high productivity provided there are no issues), the runoff detaches from the strip surface presenting a spray-like effect, named “splatter”, which is detrimental to the process.

The splattering zinc travels onto the air knives and the electromagnetic stabilisation system (EMS), where it causes poor strip surface quality and failure of the EMS respectively. Currently, there is often a delay between the occurrence of splatter and the recognition it is occurring, meaning it is difficult to identify which specific processing conditions cause the problem. Conventional manufacturing technologies would struggle to monitor and quantify the splatter phenomenon due to its complex shape and transient nature. Therefore, this paper proposes a novel computer vision (CV) application tailored for this application.

CV is the field of work concerning enabling computers to understand visual input from cameras or camera-like sensors. As a subfield of artificial intelligence, it overlaps with machine learning (ML) and deep learning (DL) whilst containing a vast array of image processing techniques. With the advent of Internet of Things (IoT) and the consequential growth of Big Data and DL, a diverse variety of CV applications are being discovered rapidly in all domains such as automotive industry with autonomous vehicles, healthcare with automated medical diagnosis, surveillance and process monitoring of manufacturing processes. With CV, tasks that require a detailed interpretation of what is visible can be revolutionised through rapid automation and enhanced performance. The CV landscape is constantly undergoing transformation through the release of new techniques and useful applications developed with said techniques.

Existing steel manufacturing applications include things like automated surface defect detection (Zhou et al., 2021), automated ladle de-slagging (Lee et al., 2021; Hao et al., 2021), automated steel section resizing (Lin et al., 2023) and automated personal protective equipment (PPE) checks (Xiong & Tang, 2021). However despite the leaps in progress, there are still countless highly beneficial applications of CV that are yet to be unearthed. Real-time monitoring of industrial operations is evidently becoming a prominent area for CV application development due to its benefits such as data-driven insights, reduced labour costs, improved quality control and process standardisation. Meanwhile, these tasks are constrained by the challenges in developing and operating models that can make inferences in real-time, the potential variability of the environment as well as the abundance of sources of visual noise in steel manufacturing sites such as poor lighting, vibrating equipment, dust, fumes and heat waves, which often confuse DL models (O’Donovan et al., 2023). Other challenges include interpretability of raw model output, operator interaction and hardware constraints. These factors collectively make developing and deploying these types of applications a difficult task but in this work, have all been addressed and overcome.

The system proposed here is a real-time molten zinc splatter severity measurement system. Primarily using the Counting (CNT) background subtraction (Zeevi, 2023) and YOLOv5 object detection (Terven et al., 2023) algorithms, the proposed device aims to revolutionise quality assurance through data-driven insights for process optimisation and predictive maintenance, as well as address a critical gap in quality control of real-time monitoring, root-cause analysis, defect detection of defects caused by excessive splatter and potentially closed-loop control depending on whether operators choose to loop the model predictions back around to the original process controls. This paper contributes in the following ways:

- It presents an innovative methodology for real-time quantification of molten zinc splatter severity that uses a novel combination of YOLOv5 for air knife detection and CNT background subtraction for splatter segmentation. This approach significantly enhances monitoring capabilities and is suitable for industrial application.
- It exhibits a novel, annotated dataset that indicates the location of air knives across sequential frames of videos that have varying environmental conditions. This is a valuable resource for further research in advancing monitoring systems and is available upon reasonable request.
- It outlines a blueprint for the deployment of a splatter monitoring system in a steel galvanising line, including details on system integration and data flow management.
- It demonstrates the potential for significant improvements in process optimisation through the application of computer vision in complex and dynamic industrial environments.

This paper is organised as follows. “Literature review" section is a literature review covering the overlap between manufacturing and CV applications. “Methodology" section describes the methodology while “Results & discussion" section presents and discusses results. “Industrial application" section addresses the model deployment and “Conclusions" section concludes the paper with key outcomes.

## Literature review

This literature review will first look at existing applications of CV in the manufacturing industry, before moving deeper into those specifically developed for steel galvanising processes. The review will then move towards existing applications of the techniques used to develop the tool; background subtraction algorithms and YOLO models. Exploring these topics will lead to exposure of gaps in research and development that will either be addressed in the remaining parts of this paper, or will be recommended as potential directions for future work.

### Manufacturing applications

CV technology has successfully been integrated with many types of manufacturing and therefore brought great benefits such as process efficiency, process quality and most importantly, process safety. This section will cover existing research on integrating CV with various types of manufacturing.

Within automotive manufacturing, CV has been used for surface quality inspection such as in Chang et al. (2020) where a quality assessment system for painted car bodies was developed as a two step process consisting of defect detection with TinyDefectRNet, a model based on YOLOv3, followed by appearance quality evaluation. The dataset was produced by splitting 200 large images into 432 patches which were then labelled (Chang et al., 2020). Recall and precision ranged from about 91.9% to 95.3% and 88.2% to 90.7% depending on whether the left, right or hood side was being analysed, whilst average analysis times ranged from 20.3s on the hood to 64.7s and 64.2s for the left and right sides respectively (Chang et al., 2020). The proposed approach for monitoring zinc splatter during galvanising also makes use of object detection for quality assessment however it is focused on directly measuring process quality rather than product quality. Another example is the use of YOLOv3 to localise and classify three types of solder joints on automotive door panels which are rectangle, semi-circle and circle solder joints (Mo et al., 2019). A dataset that consisted of 447 training samples and 106 testing samples was used to achieve a mean average precision (mAP) of 0.85 and a detection time of 0.18 seconds per panel image, which met the real-time requirements for the production line (Mo et al., 2019). This application is similar to the proposed application since both focus on meeting real-time requirements for a production line. However, the main task of (Mo et al., 2019) is classification, whereas the proposed application primarily uses background subtraction which is supported by both localisation and classification (together they constitute detection).

Electronics manufacturing also benefits from CV technology which has been shown in Zheng et al. (2021) where automated surface inspection of copper clad laminate images using defect detection was achieved through an efficient convolutional neural network (CNN) based architecture comprised of convolutional layers amongst squeeze-and-excitation blocks, as well as squeeze-and-expand blocks. A large dataset of 49560 samples was used which was split into 80% for training, 10% for testing and 10% for validation (Zheng et al., 2021). The reported precision, recall and F1 were all 0.99 and are superior to that achieved by MobileNet-v2, Inception-v3 and ResNet-50 (Zheng et al., 2021). An improved version of YOLOv3 was also used for printed circuit board (PCB) electronic component detection by using both real and synthetic data (Li et al., 2019). The real data consisted of 50 images containing 29 instrument categories such as resistors, capacitors, transformers and potentiometers, and 9145 component instances which were augmented to create a dataset 20 times the size (Li et al., 2019). The augmented dataset was split using 80% for training and 20% for testing (Li et al., 2019). The model achieved an mAP of 0.93 (Li et al., 2019). Whilst the performance metrics show these approaches to be effective, these applications focus on static images containing discrete components. Meanwhile, zinc splatter is highly dynamic and variable in terms of appearance. Also, while air knife appearance is mostly consistent, they move position which increases the complexity of the task.

There are various applications of CV in additive manufacturing such as process monitoring, defect detection and error detection. For example, one paper exhibits a hybrid CNN model architecture that was used to learn both spatial features and give a quality-level classification for a powder-bed fusion process (Zhang et al., 2020). The model was developed using 4256 training samples, 800 validation samples and 800 testing samples (Zhang et al., 2020). When tested on overheating, normal, irregularity and balling conditions the model achieved detection accuracies of 0.995, 0.996, 0.998 and 0.996 respectively (Zhang et al., 2020). These are impressive values but again, the study deals with static images and does not focus on real-time application. Another laser powder bed fusion (LPBF) paper was somewhat similar to the splatter model discussed in this paper, since the spatter signatures occurring during LPBF were segmented using a parallel model made up of a CNN and a thresholding neural network (TNN) (Tan et al., 2020). The dataset was measured in image blocks produced by splitting images into a grid format, and 5500 blocks were used for training whilst 500 were used for validation (Tan et al., 2020). Precision and recall values averaged over four different laser powers ranging from 100W to 200W were 0.777 and 0.805 respectively (Tan et al., 2020). Whilst "spatter" in additive manufacturing and "splatter" in the context of the steel galvanising line refer to different phenomena, they are similar in nature. Also, in both (Tan et al., 2020) and the proposed approach, segmentation has been used. This is because the pixel-level shape (rather than just a bounding box) is often crucial in analysing complex shapes. Interestingly, the approach in Tan et al. (2020) performs segmentation with deep learning, whereas the proposed approach uses background segmentation. This is because the splatter signatures existing in the galvanising footage are more complex than the spatter signatures seen in additive manufacturing, which highlights the novelty of this work.

Steel manufacturing has already been positively impacted by CV with an array of existing technologies being researched and developed. Similarly to other types of manufacturing, much of the progress made on developing CV applications for steel industry has been focussed around defect detection. One example of this is where YOLOv3 was used as the basis of an model developed for detecting defects on steel strip surfaces (Kou et al., 2021). The model was developed using 1800 greyscale images that were split using 90% for training and 10% for testing (Kou et al., 2021). It was evaluated against SSD300, SSD512, Faster R-CNN, YOLOv2 and YOLOv3 on two popular surface defect datasets, namely GC10-DET and NEU-DET (Kou et al., 2021). For GC10-DET, ten types of defects were localised and classified with an overall mAP of 0.713 which was the best and a speed of 45.6fps which was the second best after YOLOv2 with 51.2fps (Kou et al., 2021). For NEU-DET, six defects were detected with an overall mAP of 0.722 which was the second highest after SSD512 with 0.724 and a speed for 64.5fps which was the second highest after YOLOv2 with 127.1fps (Kou et al., 2021). Earlier in the steel lifecycle there are also various examples of CV applications, for example with slag monitoring. In terms of real-world application, steel surface defect detection could be used along the galvanising line before the zinc bath to ensure steel strip surfaces are suitable before coating, as well as after the air knives to ensure the strip has been coated properly. However, the proposed approach addresses splatter severity which immediately indicates the quality of the coating process, which has not been found in any other published work. In Kim et al. (2020), a CNN was used to predict the optimal slag removal path during de-slagging of ladles which was then intended for use with a robot to automate the de-slagging task (Kim et al., 2020). Similarly, to Tan et al. (2020), the model was trained using 1568 blocks and tested using 1046 blocks. Testing accuracy of over 91% was achieved for the CNN and overall the slag removal path was estimated with approximately 90% accuracy (Kim et al., 2020). Both the work in Kim et al. (2020) and the work proposed here move towards automating operator behaviour when carrying out a task. However, whilst the work in Kim et al. (2020) intends to simply automate the task, the work proposed here intends to surpass the observational capabilities offered by an operator by continuously providing quantitative, objective and precise results multiple times per second.

### Background subtraction applications

Background subtraction is the name given to the set of techniques used for efficiently segmenting foreground pixels from background pixels in a sequence of images. Whilst the deep learning CV task of segmentation is similar in nature to background subtraction, they have clear differences such as the complexity of the algorithms, how they learn features as well as their capabilities.

For this application, it was decided that it was more appropriate to use background subtraction rather than a segmentation network such as Mask R-CNN (He et al., 2017) or YOLACT++ (Bolya et al., 2022), since these models require large labelled segmentation datasets which when observing the air knife footage, would be almost impossible to label accurately within a practical amount of time due to the finite form the splatter sometimes presents itself in. Not only this, but deep learning models can sometimes be difficult to operate in real-time without spending large amounts of time optimising the model and its deployment approach.

Oppositely, background subtraction algorithms, particularly those available in OpenCV, are capable of segmenting the background with great detail within just a few sequential frames and zero labels. Furthermore, the algorithms on their own can make inferences in real-time with little to no optimisation. Of course, there are limitations such as the inability to specify what kind of objects are segmented and the inability to deal with a moving camera. This means background subtraction is particularly well-suited for scenarios where the background is static and the object of interest is dynamic, such as in the air knife region.

Recent advancements in image segmentation, such as the Segment Anything Model (SAM) (Kirillov et al., 2023), the FastSAM (Zhao et al., 2023) variant and Robust Saliency-aware Distillation (RSaD) (Liu et al., 2024) exemplify significant developments in the field and could potentially be integrated into future iterations of splatter severity systems. SAM is a segmentation network with zero-shot capability, meaning it can segment objects without being trained for specific objects (Kirillov et al., 2023). This is promising for the research project discussed in this paper, however currently SAM lacks the real-time processing required for dynamic environments such as those found in steel galvanisation. Whilst SAM is reported to take from 110ms to 5147ms to process one image depending on complexity, FastSAM is reportedly capable of operating at 40ms per image which is much more applicable (Zhao et al., 2023). However, both models require explicit input prompts which adds unnecessary complexity when deploying them in fully automated systems where minimal human interaction is desirable (Kirillov et al., 2023; Zhao et al., 2023). Considering these factors, whilst SAM and FastSAM significantly advance the field of computer vision, they are currently unsuitable for real-time automation applications in dynamic industrial environments. Additionally, the RSaD method is a recent advancement in terms of enhancing segmentation of fine-grained features and is considered few-shot since it is only trained on approximately one to five samples per class (Liu et al., 2024). These aspects could be beneficial for achieving robust segmentation of complex industrial processes with minimal annotated data requirements, however RSaD has high computational demands due to its fine-grained nature which is detrimental for application to high-speed production lines (Liu et al., 2024).

OpenCV algorithms such as mixture of Gaussians (MOG), MOG2 and the Gaussian mixture-based background foreground segmentation algorithm (GMG) are Gaussian mixture models (GMM), meaning they subtract the background using a combination of Gaussian probability densities. In other terms, a set of Gaussian distributions each represent part of the background which combine to represent the entire background (Zivkovic, 2004). Literature exists on attempts to combine deep learning with background subtraction such as Machado et al. (2021); Christiansen et al. (2016); Yu et al. (2019). Combining techniques from the two types of algorithms is a potential area of future research that could produce exciting results for industry.

Whilst background subtraction has not yet been applied to this kind of application, it has still been applied successfully for various use cases. For example, one paper proposes the use of the MOG background subtraction algorithm in combination with a timed motion history image (motion segmentation) method and Kalman filtering as part of a real-time vehicle traffic tracking system (Qu et al., 2010). Another paper evaluates all of the available OpenCV algorithms with the task of ship detection on inland waters and finds that the GSOC (Google summer of code) and CNT algorithms are the best for that particular application (Hyla et al., 2019). One final example is a comparison of the GMG, KNN (K-nearest neighbours), MOG and MOG2 algorithms at performing subtraction on near infrared spectrum images of moving wild mammals for animal detection (Trnovszký et al., 2017). The study found that when evaluating algorithms using handcrafted labels, the KNN algorithm mask was most similar to the labels, followed by MOG2 in terms of similarity but MOG2 was faster and therefore more suitable for real-time processing. These examples demonstrate just how versatile background subtractions algorithms are for tracking moving objects. The proposed approach however, advances on these applications firstly in that the structure of the splattering zinc is far more complex and variable than the objects in these examples (road vehicles, ships and animals). Secondly, the proposed approach analyses segmentation masks in real-time for deeper insights. Additionally, by integrating object detection, this work enhances the robustness and precision of motion detection.

Examples of background subtraction use for manufacturing purposes firstly include (Nettekoven et al., 2022) where over 30 different segmentation algorithms (including OpenCV background subtraction algorithms) were evaluated when applied to infrared laser track images in LPBF. The results showed that despite struggling to segment laser tracks, MOG, MOG2, CNT, GSOC and KNN were the only algorithms able to exclude spatter from the foreground, suggesting they were more robust to spatter-like noise (Nettekoven et al., 2022). The ability to distinguish between spatter-like structures and other signals is crucial for monitoring zinc splatter, and the results from (Nettekoven et al., 2022) suggest that the mentioned algorithms may be beneficial. The MOG algorithm was applied in Bonello et al. (2020) for inspection of missing and misaligned components in printed circuit board assemblies (PCBAs). The results showed the algorithm was capable at distinguishing between reference PCBA images and a defective PCBA images, highlighting the effectiveness of the method (Bonello et al., 2020). Whilst the study in Bonello et al. (2020) is innovative and effective, it focuses on still images and essentially performs anomaly detection for quality control. The approach here is different because although it still contributes to quality control, it involves continuously monitoring a dynamic process, and is also beneficial for process optimisation. The CNT algorithm was used in Sabih et al. (2023) to distinguish raw materials on a conveyor belt system from the background, in order to monitor material flow rate on a soda-ash production line. In combination with a frame difference technique, the CNT algorithm proved to be effective in precise, real-time analysis required for process optimisation for improved gas production, reduced production waste and reduced costs (Sabih et al., 2023). Similarly, the CNT algorithm has been used in this work to precisely monitor zinc splatter in real-time to improve steel strip quality, reduce waste and costs, and minimise equipment downtime. Again, the dynamic splatter structure in this work is far more variable and complex than the raw materials in Sabih et al. (2023), emphasising the advancement made by this study.

### YOLOv5 applications

YOLOv5 is the fifth version of the you-only-look-once object detection models and is comprised of three parts. Firstly, CSP-Darknet53 is used as the CNN backbone which essentially performs feature extraction (Terven et al., 2023). Secondly, spatial pyramid pooling (SPP) and path aggregation network (PANet) perform pooling and feature aggregation respectively, which is considered the neck of the architecture (Terven et al., 2023). Finally then, the prediction head predicts bounding boxes, class probabilities, and objectness scores (Terven et al., 2023). YOLO models are possibly the most widely used and well-known object detection models existing today, with at least eight versions at the time this paper was written. A few YOLOv3 applications have already been mentioned in the manufacturing applications section. YOLOv5 is one of the most suitable choices for applications due to its speed, flexibility, active open source community, user-friendly implementation and general ease of deployment. Whilst it is recognised that YOLOv7 and YOLOv8 are now available and suitable for application with good support, YOLOv5 has more information built up by community contributions over time.

Some notable YOLOv5 applications include safety helmet detection which was achieved by replacing the conventional non-maximum suppression (NMS) with DIoU-NMS and using 6000 images for training and 1000 for testing, achieved an average precision (AP) of 0.957 at 98fps (Tan et al., 2021), tomato virus disease recognition that used 1036 samples split using 80% for training, 10% for testing and 10% for validation of YOLOv5 with an additional squeeze-and-excitation module, achieving 0.868 precision, 0.922 recall and 0.760 mAP_COCO_ (Qi et al., 2022), and forest fire detection using a model based on YOLOv5 with changes such as from the Spatial Pyramid Pooling-Fast (SPPF) module to the Spatial Pyramid Pooling-Fast-Plus (SPPFP) module, addition of a convolutional block attention module (CBAM) and changing the PANet to a bi-directional feature pyramid network (BiFPN) (Xue et al., 2022). The forest fire study used 3170 data samples split using 80% for training, 10% for testing and 10% for validation, and experiments showed that with all of the aforementioned modifications to YOLOv5, an mAP_0.50_ of 0.821 was achieved for forest fire detection with a speed of 54.1fps (Xue et al., 2022). Meanwhile, the original YOLOv5s only achieved mAP_0.50_ of 0.761 with a slightly higher speed of 55.2fps (Xue et al., 2022). These studies emphasise how YOLOv5 can be modified to perform well in a variety of scenarios in terms of both precision and speed. The proposed approach uses YOLOv5 as a secondary technique to support the primary technique of background subtraction, and since the air knives remained mostly unchanged, there was no need to modify the YOLOv5 architecture. However, real-time quantification of zinc splatter drove the need for combining YOLOv5 with background subtraction which expands current capabilities in the field.

Examples of YOLOv5 in manufacturing specifically firstly include (Le et al., 2022) where it was used for surface defect detection of micro-motors on the assembly line, which were classed as either normal, dirty, structurally distorted, deformed at the main body or incomplete. Using a total of 1400 labelled images and 8613 bounding box labels which were split so 80% were used for training and 20% were used for validation, YOLOv5 achieved an mAP of 0.734 and an inference time of 6.4ms (Le et al., 2022). The study presented in Zendehdel et al. (2023) optimised YOLOv5 for real-time detection of detection of tools used in smart factories. In Zendehdel et al. (2023), 3286 images of 17 different classes of tool were split using 70% for training, 15% for testing and 15% for validation and the model achieved an mAP of 0.983 with no mention of the final inference speed other than describing the model as real-time. Finally, (Chen et al., 2023) presents the application of YOLOv5 modified for weld type classification, tacked spot recognition and weld region of interest determination for robotic welding. A total of 3450 structured light images from different welding assemblies were used for model development, with a 65% used for training, 20% used for validation and 15% used for testing (Chen et al., 2023). The overall method was shown to achieve 100% precision and recall and an inference time of 18ms (Chen et al., 2023). Manufacturing applications of YOLOv5 presented here again show the efficacy of the model in various scenarios which suggests it is a versatile tool with high performance in terms of both speed and precision. In comparison, the front faces of the air knives are basic compared to tools and motors (however they do move along multiple axes), but the underside faces of them are more complex and only appear depending on the camera position. Therefore, this work involves the training of YOLOv5 for object instances that may not exist for the entire video, which is unique when looking at the literature. Also, in this work, detection is applied purely to video footage (a series of frames) as opposed to one image at a time.

There are some key findings from reviewing the literature which are as follows. CV is suitable for a large range of applications in different types of manufacturing such as automotive, electronics, additive and steel, and across these the majority of CV developments are based on defect detection and inspection systems. Meanwhile, there is no published work on real-time monitoring of molten zinc splatter occurring on the galvanising line, meaning this work addresses a novel challenge. In fact, due to CV with deep learning being an emerging field, there are many gaps in the literature. For example, many studies achieve high performance but have no focus on real-time application. Even for technology that is not on the processing line, speed and efficiency are paramount in industry and therefore real-time capabilities are crucial in advancing the field. Additionally, many studies use object detection alone to perform tasks where adding a segmentation component would be greatly beneficial. Furthermore, in manufacturing there are many existing processes that could benefit from CV that have not been addressed at all. The splatter monitoring system developed in this work uncovers a significant type of use case for CV in manufacturing, which is to monitor variables that are visible but not currently monitored due to the lack of compatibility with traditional manufacturing technology. Examples of other applications could be quantifying the light intensity during arc welding for process quality and process safety, quantifying the quality of welded joints or assemblies, quantifying the visible degradation of equipment for maintenance planning, monitoring of process temperature distributions from a distance to prevent the need for degradation of measurement tools, and many more.

This work also highlights the difference between background subtraction and object segmentation. Background subtraction algorithms are much easier to train than neural networks that perform segmentation on objects they are trained for long periods of time to recognise, however are normally only suitable for static backgrounds. Both methods have advantages and disadvantages, and combining background subtraction and DL algorithms could be a great way to advance the fields of CV and manufacturing.

Finally, the versatility of YOLOv5 and other variants as tools for robust real-time object detection has been demonstrated. The integration of YOLO models with other techniques for tackling real-world challenges has been exemplified through literature and promoted through discussion, which contributes to the advancement of the field.

## Methodology

**Fig. 2:**
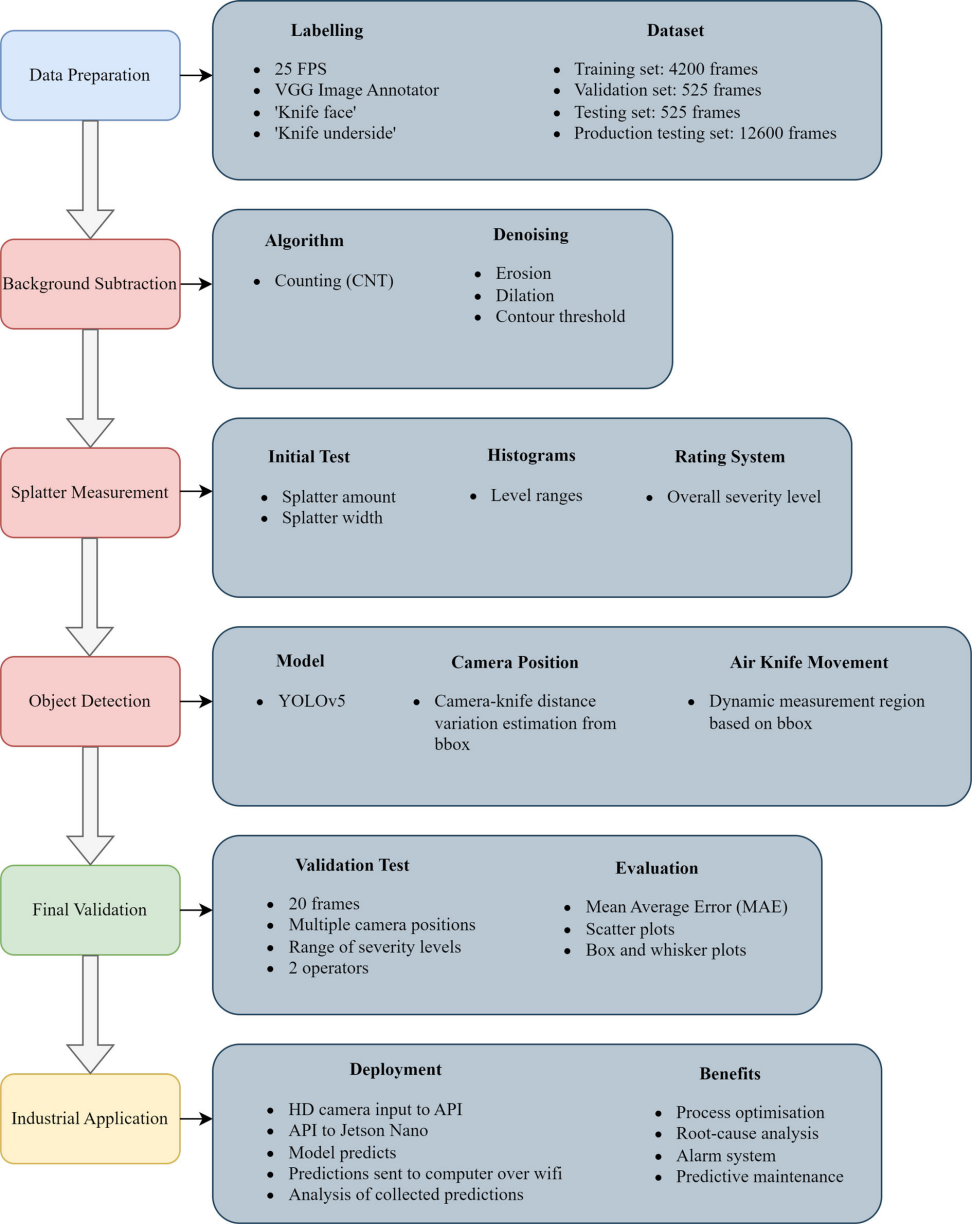
An overview of the methodology used for this work

The overall task for this body of work was to develop a tool that can in near real-time, quantify the severity of molten zinc splatter occurring along a steel galvanising line at high strip speeds due to air knives that wipe off excess zinc to give a uniform thickness coating along the steel strip. Using this tool, the operators at the coating site can collect data on the severity of splatter whilst using different settings for process parameters such as strip speed, air knife distance and air knife pressure, and then find relationships between the two sets of data which can be used to optimise the galvanising process to minimise splatter severity whilst maximising strip speed. The methodology followed is summarised in Fig. 2.

The flow chart has six steps. Firstly, data preparation began with acquiring footage from a local galvanising site that showed the air knives, steel strip and splatter occurrences whilst the site was in operation. Once the footage was acquired it was divided into frames for processing, which was sufficient for the background subtraction stage but not object detection. Therefore, it was then necessary to label the data which was done using VGG image annotator (VIA) (Dutta et al., 2016).

The prepared data was used for the second step of running different background subtraction algorithms to find the best choice for the splatter model. The parameters of the best algorithm were then adjusted through a methodical trial-and-error process which focused on optimising the trade-off between sensitivity to splatter detection and robustness against noise from environmental factors like heat and dust. Background subtraction was used to segment pixels that represented splattering zinc from the background pixels. These algorithms adapt to scene changes over time and generally perform well in scenarios with static backgrounds. Since the aim was to achieve real-time processing and adaptability to erratic splatter patterns in an environment with a mostly consistent background, these algorithms were suitable for this application.

The third step used the output mask from background subtraction to properly quantify the splatter severity. It was quantified in terms of splatter amount (the number of pixels in the designated splatter region) and splatter width (how widespread the splatter was in terms of pixels). These values were plotted as histograms then used to give an overall splatter severity rating using a proposed rating system.

The fourth step was integration of object detection during which YOLOv5 was used for two purposes. Firstly, to ensure the model could deal with vertical and horizontal air knife movement. Since background subtraction techniques are not good at dealing with dynamic backgrounds and the relevant region of splatter was always below the air knives which sometimes moved, detecting the air knives with YOLOv5 identified the region for measurement and ensured it was adjusted dynamically. Secondly, cameras were moved by operators between shifts and therefore did not have a consistent viewpoint. Using YOLOv5, detection box sizes could be used to indicate the distance between the camera and the air knives and therefore scale the background subtraction outputs depending on this distance. Overall, YOLOv5 ensured the model was able to adapt to a changing environment in both real-time and long-term cases.

The fifth step was model validation where comparisons were made between the model developed in this paper, and the judgement of operators observing the splatter. Mean average error (MAE), scatter plots and box and whisker plots were used to assess results.

The final step is the model deployment strategy which has been presented in this paper as a future plan rather than a completed task. Deployment in itself is a substantial task and therefore an overview has been presented rather than a full report. This includes a workflow and the benefits of deployment.

The method used in the third step of Fig. 2 for measuring the severity of splatter using quantity and width, is a novel contribution the the field that could be applied to industrial monitoring applications. Also, there is novelty in the combination of a background subtraction algorithm with an object detection algorithm for improved adaptability and reliability of real-time manufacturing process monitoring. This approach sets a foundation for future automated industrial quality control.

**Fig. 3:**
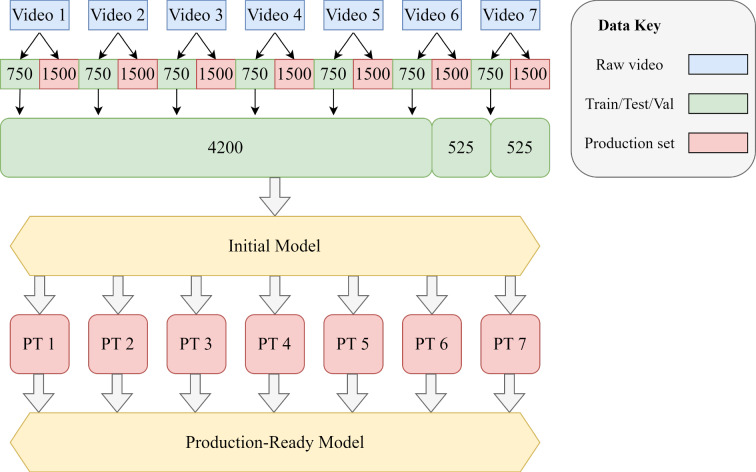
Diagram showing overview of data strategy

**Table 1:** Description of videos and their settings

Name	Splatter	Air Knife Movement	Camera Position
Video 1	Entire range	No	Normal distance
Video 2	Low Mid	No	Close to knives
Video 3	Low	No	Far from knives
Video 4	Low	No	Zinc pool shown
Video 5	Low	Horizontal	Angled towards right
Video 6	Entire range	No	Angled towards right
Video 7	Low-Mid	Vertical	Close to knives

### Data preparation

An overview of the data strategy used for this work is shown in Fig. 3, whilst descriptions of each source video is provided in Table 1. Within VIA, for every frame, both front faces of the air knives were labelled with a bounding box as “Knife Face” whilst Underside faces of the knives were also labelled but as “Knife Underside”. This was done for 30 seconds of seven different videos (Video 1 to Video 7) with different viewpoints and process conditions. Since the footage ran at 25fps, this meant 750 frames for each video were prepared, giving a model development dataset of 5250 samples. The dataset was divided by using approximately 80% (4200) for training, 10% (525) for validation and 10% (525) for testing as shown in green in Fig. 3. Furthermore, seven one-minute videos (shown in red) were used for some further production testing (PT) of the model to ensure the prototype was fit-for-purpose before attempting deployment and so were considered part of the validation stage before considering the prototype complete. The pieces of footage were originally from the same videos as the seven sets of 30 second videos used for the original dataset. Once all required data was prepared for object detection the next step was to look at background subtraction algorithm selection.

### Background subtraction

Background subtraction is the task achieved by a certain set of algorithms designed to separate background pixels from foreground pixels across frame sequences. The algorithms tested were MOG, MOG2, LSBP (local singular value decomposition binary pattern), GSOC, GMG, KNN and CNT.

Of these, MOG, MOG2, GMG are Gaussian-based (briefly mentioned in the literature review). Gaussian distributions (also called normal distributions) are essentially bell curves that are symmetrical and are typically defined by their mean ($\mu$) and variance ($\sigma {^2}$) (Newcastle University, 2024). Gaussian distributions are commonly used to model the distribution of values in various forms of data, including images and videos, where they are often used for smoothing (OpenCV, 2023b). Gaussian mixture models operate by modelling pixel values over a series of frames as a mixture of Gaussian distributions to emphasise associations between groups of pixels whilst allowing for small variations such as changes in lighting and shadows (Zivkovic, 2004). Equation 1 calculates the probability that a certain pixel has a value of $x{_N}$ at time *N*, where each pixel is modelled by a mixture of *K* Gaussians (KaewTraKulPong & Bowden, 2002).

1 $$\begin{aligned} p({\textbf{x}}_N) = \sum _{j=1}^{K} w_j \eta ({\textbf{x}}_N; \theta _j) \end{aligned}$$

In Eq. 1, $w{_j}$ is the weight parameter of the j^th^ Gaussian component, $\eta$ is the probability density function and the j^th^ Gaussian component parameters including mean and covariance are represented by $\theta _j$ (KaewTraKulPong & Bowden, 2002).

Each component captures part of the background and collectively their weights indicate the likelihood of different pixel values (Zivkovic, 2004). When each new frame is processed, pixels that do not align with the expect background pixels are considered foreground pixels (Zivkovic, 2004). In GMMs, the number of Gaussians for each pixel plays a key role in performance outcomes (Zivkovic, 2004).

Alternatively, the LSBP algorithm is based on SVD binary patterns and despite GSOC not having a dedicated paper, it is a successor of LSBP (Guo et al., 2016), (OpenCV, 2023a), (Bobulski, 2022). LSBP is a combination of local binary patterns (LBP) and SVD. LBP captures textural information by comparing each pixel with its neighbours and encoding these relationships into a binary pattern (Ojala et al., 2002). Equation 2 can be used to calculate LBP based on *P* neighbouring pixels at radius *R*, where *s* is the sign function used to compare the intensity of the central pixel to that of the neighbouring pixel, $g{_p}$ is the intensity value of the p^th^ neighbouring pixel, $g{_c}$ is the intensity value of the central pixel being evaluated and $2{^p}$ is the binomial factor which corresponds to the p^th^ neighbour’s position (Ojala et al., 2002).

2 $$\begin{aligned} LBP_{P,R} = \sum _{p=0}^{P-1} s(g_p - g_c)2^p \end{aligned}$$

However, LBP is not robust to local image noise when neighbouring pixels are similar (Guo et al., 2016). Therefore SVD, which is used for dimensionality reduction of rectangular matrices, is integrated with LBP to enhance robustness by emphasising the most significant patterns within the data which reduces the effect of noise and results in better background stability (Guo et al., 2016). Equation 3 can be used to perform SVD on a matrix *B* surrounding the location (*x*, *y*), where *U* and *V* are orthogonal matrices, and $\Sigma$ is a diagonal matrix containing the singular values of *B*(*x*, *y*) (Guo et al., 2016).

3 $$\begin{aligned} B(x, y) = U \Sigma V^T \end{aligned}$$

LSBP works by firstly computing the LBP descriptor for each pixel using local neighbourhoods, secondly by creating matrices of the descriptors, thirdly by applying SVD to the matrices to obtain principal components to reduce noise, and finally by using the components to robustly identify the foreground and background (Guo et al., 2016). Equation 4 calculates the LSBP binary string at $(x{_c},y{_c})$, where $i{_p}$ is the neighbourhood point value and $i{_c}$ is the central point value (Guo et al., 2016).

4 $$\begin{aligned} LSBP(x_c, y_c) = \sum _{p=0}^{P-1} s(i_p, i_c)2^p \end{aligned}$$

KNN background subtraction is based on the common machine learning technique called K-nearest neighbours, which is used for classification based on feature similarities (Scikit-learn Developers, 2023). In the context of background subtraction, the KNN algorithm is a non-parametric technique that uses a kernel to classify pixels as belonging to the foreground or background (Zivkovic & van der Heijden, 2006). The kernel is described as a "balloon estimator" and the diameter of it is dynamically adjusted to cover a predefined number of data points which varies depending on the density of local data (Zivkovic & van der Heijden, 2006). The "density" of data refers to the extent of similarity between pixels in terms of features such as colour (Zivkovic & van der Heijden, 2006) This approach means the KNN algorithm can effectively adapt to areas of varying sample density, making it robust to noise and capable of handling gradual background changes (Zivkovic & van der Heijden, 2006). Equation 5 shows the formula for the non-parametric density estimate which distinguishes between background (BG) and foreground (FG) components (Zivkovic & van der Heijden, 2006). In Eq. 5, *T* is the number of historical frames used for adaptation, *t* is the current time, *m* is the earliest frame that the algorithm begins to iterate through until it reaches *t*, $\overrightarrow{x}^{(m)}$ is the pixel RGB value at time *m*, $\overrightarrow{x}$ is the pixel RGB value at the current time, *k* is the number of samples from the dataset $X{_T}$ that lies within the hypersphere (balloon) volume *V* of the kernel which has diameter *D*, and the kernel function is denoted by ${\mathcal {K}}(u)$ (Zivkovic & van der Heijden, 2006).

5 $$\begin{aligned}{} & {} {\hat{p}}_{\text {non-parametric}}(\overrightarrow{x} | X_T, BG+FG)\nonumber \\{} & {} \quad = \frac{1}{TV} \sum _{m=t-T}^{t} {\mathcal {K}} \left( \frac{\Vert \overrightarrow{x}^{(m)} - \overrightarrow{x}\Vert }{D} \right) = \frac{k}{TV} \end{aligned}$$

The CNT algorithm is another non-parametric approach that counts the number of frames each pixel has remained constant for, and uses pixel stability values to decide on whether the counted value of each pixel should mean it is foreground or background (Zeevi, 2023). There is also a threshold to decide on the boundaries for what is considered the same colour (Zeevi, 2023). It is not based on any particular distributions and therefore is not represented by a formula. However, Algorithm 1 captures the essence of the CNT algorithm.

**Algorithm 1:**
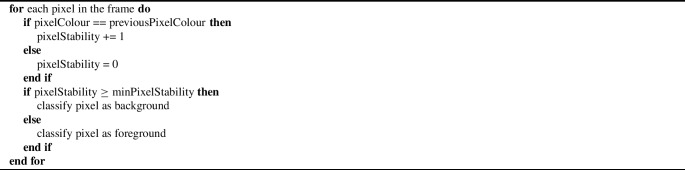
CNT Algorithm

All algorithms are available in OpenCV and were implemented using Python. Each algorithm has its own set of parameters that can be changed, and some have no changeable parameters. The steps listed next were taken to decide on the best algorithm for the splatter model.

- Process Video 1 using algorithm.
- Record speed and observe precision.
- Eliminate algorithms slower than 1fps.
- Optimise best overall model for air knife movement using trial-and-error.

This approach was taken so that firstly, time was not wasted optimising all models for air knife movement. Secondly, efficiency was further practiced through the elimination of slow algorithms. Overall this was resource-efficient and ensured that the speed and accuracy requirements for the application were met. For optimisation, trial-and-error was used over an alternative such as grid search, since there was already some inclination as to what the values should be based on previous results with default parameter settings. Values were adjusted incrementally to balance sensitivity to splatter dynamics with resistance to noise from environmental factors such as heat distortion, dust and air knife movement. Since parameters were specific to each algorithm, more information is provided in “Results & discussion" section.

### Splatter severity measurement

**Fig. 4:**
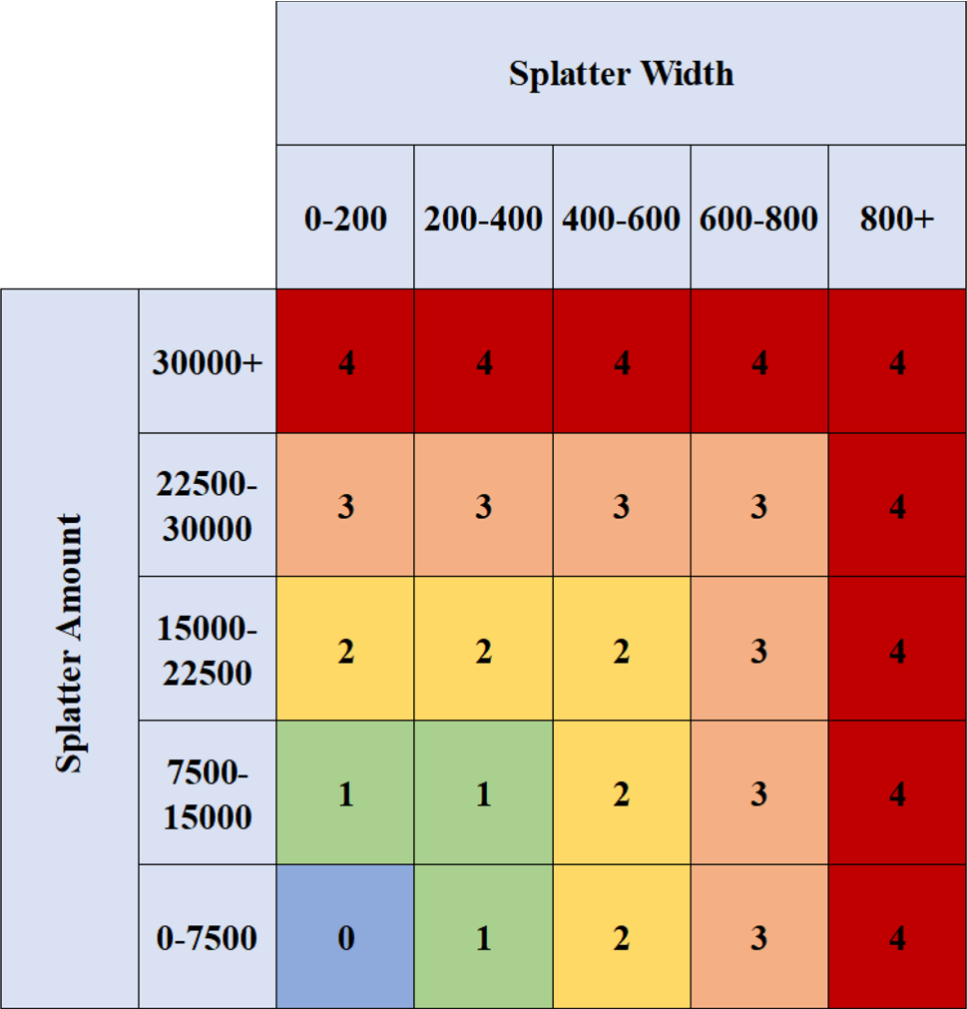
The rating system used to obtain an overall splatter severity rating

The optimal background subtraction algorithm gave a mask output that could be used for splatter measurement and this was processed using erosion and contour thresholding for denoising. Erosion is when the outer boundaries of contours are thinned. In this work, erosion was performed with a (2,2) filter to eliminate noise due to camera shaking and faint heat waves visible in the footage. The contour threshold ensures the model ignores contours within the splatter region that are below 75 pixels in size in order to remove some noise that remained after erosion noise removal. Only the pre-processed output within the splatter measurement region was used and this was observed for two features; splatter width and splatter amount, where both are measured in pixels. These were recorded for every frame and plotted on two separate histograms which were used to heuristically choose five different ranges representing five different severity levels for splatter amount and splatter width. Based on the individual severity levels for splatter amount and width, an overall splatter severity rating was given using the rating system shown in Fig. 4. The system works by the assumption that splatter amount and splatter width contribute equally to the overall severity rating, and therefore the overall severity rating will be equal to the highest of the two values.

### Object detection

After splatter severity was quantifiable, it was necessary to make the model robust to variations in camera position. This was achieved using object detection for detecting air knives, which was also beneficial for automatically defining the splatter measurement region (shown in “Results & discussion" section) while the air knife position and scale moved due to camera perspective and vertical and horizontal air knife movement.

Object detection was achieved using YOLOv5. The technical details of YOLOv5 were introduced in the literature review. To reiterate briefly, the CNN backbone takes images as input and performs feature extraction, the SPP and PANet pool and aggregate extracted features and then the prediction head predicts bounding box co-ordinates, class probabilities and objectness scores (Terven et al., 2023). As with all supervised detection networks, YOLOv5 required training, validation and testing stages. The model was trained for 30 epochs on YOLOv5s using 4200 training samples and 525 validation samples which were used at the end of each epoch to monitor how well the model is generalising to unseen data. Finally, the remaining 525 testing samples were used to give a more accurate indication of how well the final model would generalise on unseen data.

Once object detection was successful, the bounding box predictions were used to redefine the splatter measurement region. Bounding boxes were then used to estimate the distance between the camera and the air knives relative to other camera positions present in the originally acquired footage. The relative distance was represented as a scaling factor (SF) and calculated as shown in Eq. 6, where B represents the current bounding box size and R represents the reference bounding box size.

6 $$\begin{aligned} \textrm{SF} = \frac{\textrm{B}}{\textrm{R}} \end{aligned}$$

The scaling factor was calculated per frame which was sub-optimal as the bounding box sizes vary slightly between every frame and the camera position normally stays constant for hours at a time. Therefore, a moving average (MA) of the scaling factor was calculated every frame and used as the final value for the model. The equation is shown in Eq. 7 where X represents the scaling factor value calculated for one frame and n represents the number of points averaged.

7 $$\begin{aligned} \mathrm{MA(SF)} = \frac{X_{1} + X_{2} +...+X_{n}}{n} \end{aligned}$$

### Expert validation

Upon nearing model completion, it was important to validate the functionality and performance of the model to ensure it was developed appropriately for deployment. Firstly, the seven one-minute videos were processed by the model and the seven output videos were analysed by eye to ensure the model appeared to be functioning properly on different potential inputs it may need to handle during real-world application. Secondly, a validation test was produced and completed by two operators at the galvanising site. The test consisted of operators estimating the splatter severity of 20 frames with various camera and process conditions and then comparing estimations to the ratings given by the model.

## Results & discussion

The proposed approach introduces a novel application of technology to monitoring zinc splatter severity. At the time of writing, there are no directly comparable studies documented in the literature. This is one of the key contributions of the work since it addresses this gap.

### Background subtraction

The first set of results were from the initial testing of different background subtraction algorithms to find which was most appropriate for the task. Video 6 alone was used to evaluate algorithms since differences in performance were obvious enough to not require testing on other sample videos. Also, brief testing showed differences in camera position did not affect performance significantly. However, in Video 7 where the air knives move upwards, the system appearance changed which impacted algorithms and is addressed later. The first performance metrics observed were inference time and consequential speed which are shown in Table 2. As shown in bold, CNT algorithm inferences were much faster than inferences made by any other algorithm, which meant it also performed at the highest frame rate.

**Table 2:** Inference Times and Frames Per Second for Various Algorithms

Algorithm	Inference Time (s)	Frames Per Second
MOG	0.0951	10.5152
MOG2	0.1116	8.9606
LSBP	1.0188	0.9815
GSOC	0.4066	2.4594
GMG	0.2733	3.6590
KNN	0.1286	7.7760
**CNT**	**0.0505**	**19.8020**

**Fig. 5:**
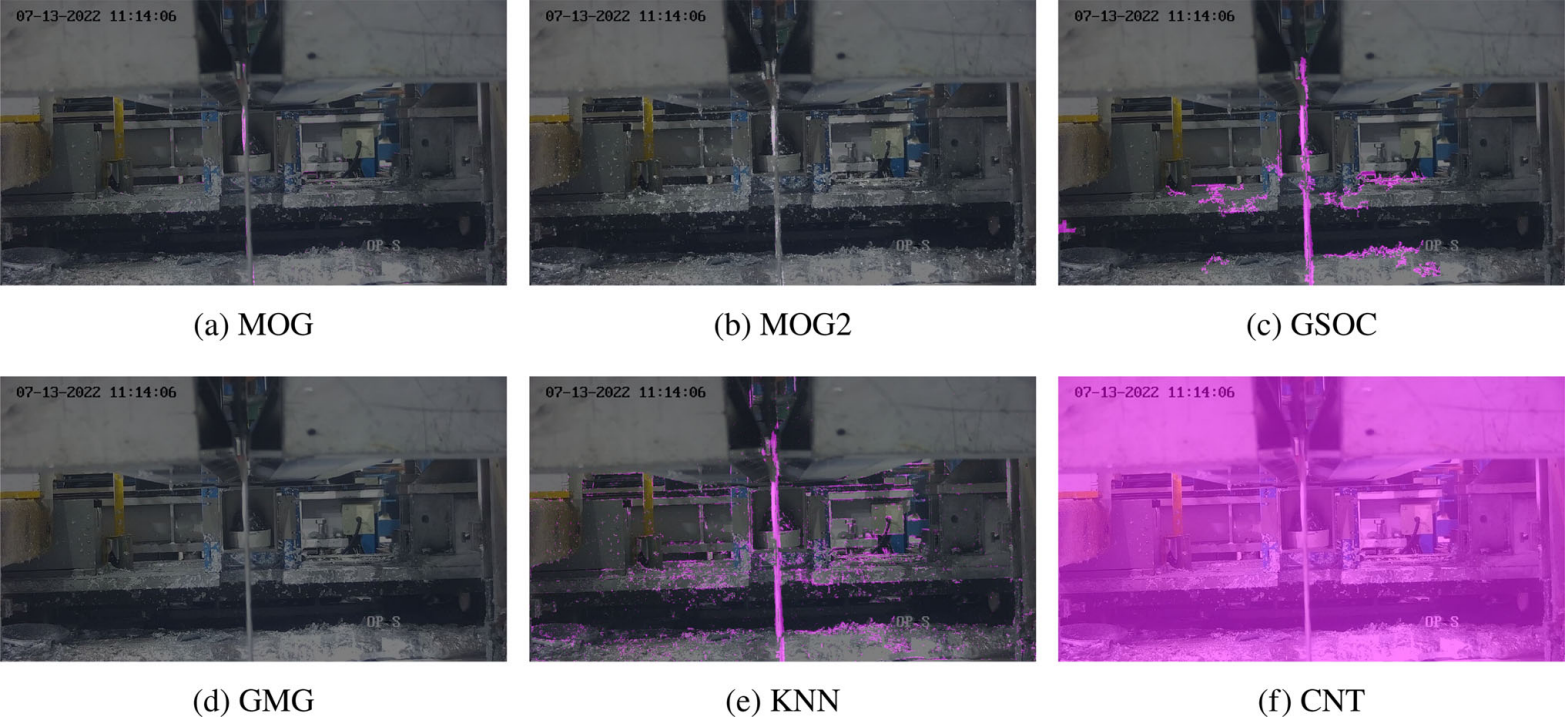
Effects of different background subtraction algorithms on an early frame with low splatter

The raw footage initially acquired ran at 25fps and so this should he considered “real-time” performance. The target was to reach “near real-time” performance so that most frames of the original footage would be processed during deployment. Therefore, considering that YOLOv5 inference, splatter measurement and other steps would also be performed for every frame, the speed of the chosen algorithm needed to be as high as possible. Whilst acknowledging this, the frame rates shown in Table 2 can be increased significantly in multiple ways such as using a GPU, resizing frames and optimising algorithm parameters. The values should therefore be evaluated relative to each other and not based on whether they run at 25fps or not. That said, it is clear from Table 2 that the algorithms initially appearing most suitable for real-time monitoring are MOG, MOG2 and CNT, whereas the least suitable is LSBP. Based on the results, LSBP was eliminated from the process and no longer considered for the final model.

Figure 5 shows a frame during the first second of footage processed by each of the algorithms (excluding LSBP) with default parameter settings. In this frame there is what could be describe as “low” severity of splatter. An initial observation from the output video showed that the GMG algorithm segmented nothing, MOG2 started to track pixels shown in grey but were not fully segmented, MOG segmented a small amount of zinc, GSOC and KNN segmented zinc and background noise, and CNT required a certain number of frames to “learn” where the background was, so had not yet initiated.

**Fig. 6:**
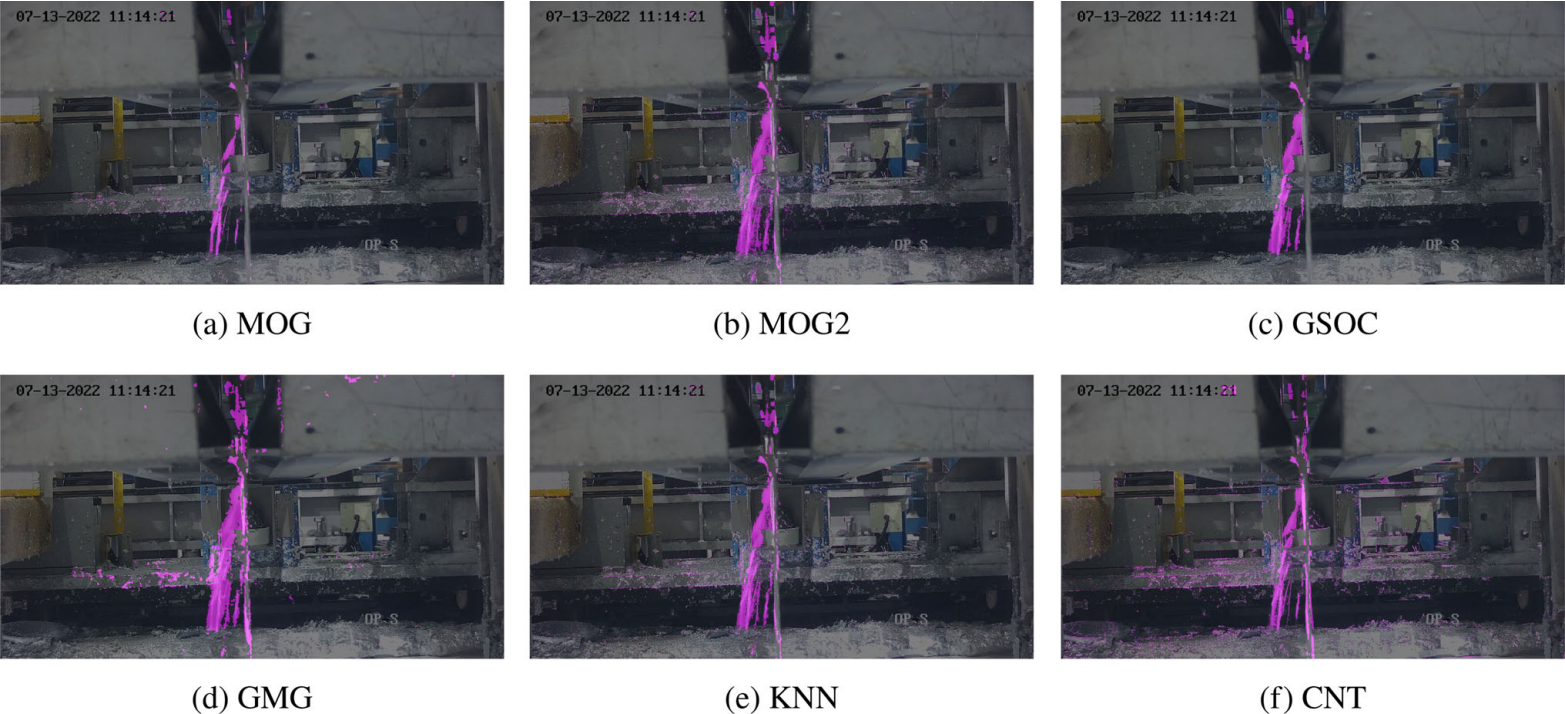
Effects of different background subtraction algorithms on a frame with moderate splatter

Figure 6 shows a frame during the 18th second of footage processed by each of the algorithms except LSBP with default parameter settings. In this frame there is what could be describe as “moderate” severity of splatter. CNT followed by MOG segmented the splatter most precisely, whilst all other algorithms segmented the regions between streams of splatter. The CNT algorithm was most precise at segmenting the splatter however also carried some background noise, whereas MOG carried significantly less.

**Fig. 7:**
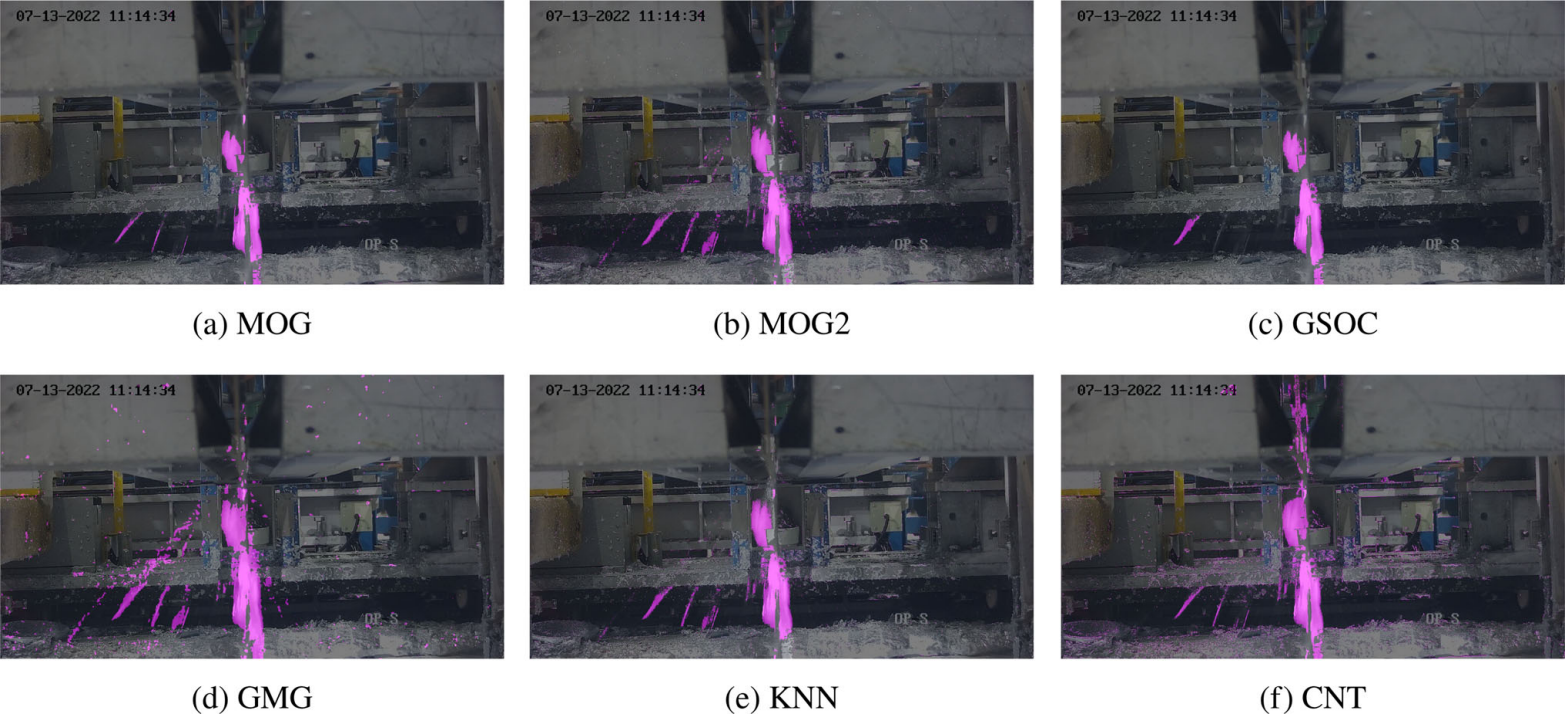
Effects of different background subtraction algorithms on a frame with high splatter

Figure 7 shows a frame during the 33rd second of footage processed by each algorithm except LSBP with default parameter settings. There is what could be describe as “high” severity of splatter. GSOC did not segment enough pixels whereas GMG segmented too many. All other algorithms performed well with CNT producing noticeably more background than the others. Overall, the CNT algorithm was most suitable since it had a significantly higher frame rate, was close to real-time, and was the most precise during testing. The limitations of CNT were the initial learning stage which took about 15 frames and was not an issue since the model is expected to take some time to initialize, and the background noise which can be eliminated using erosion which is discussed later.

**Fig. 8:**
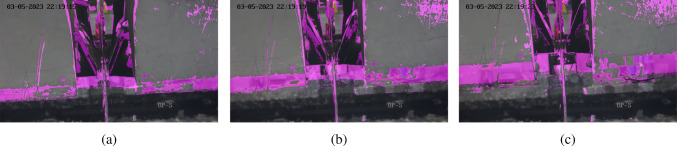
Effects of knife movement on CNT background segmentation

The next step was to ensure it could deal with the air knife movement in Video 7. Therefore, the algorithm processed the video with default parameter settings. Three frames at the 12th, 16th and 21st second marks are shown in Fig. 8, which show there is an issue. Since the algorithm has defined the background over hundreds of frames prior to knife movement, when the knives move it is detected by the CNT algorithm which makes the segmentation mask inaccurate. This inaccuracy can be seen increasing across the frames in Fig. 8.

**Fig. 9:**
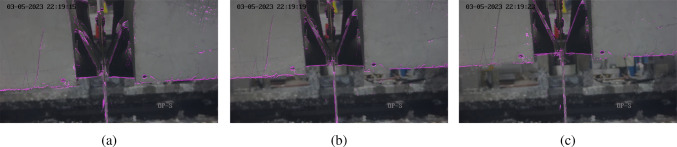
Effects of knife movement on CNT background segmentation after modifying CNT algorithm

**Fig. 10:**
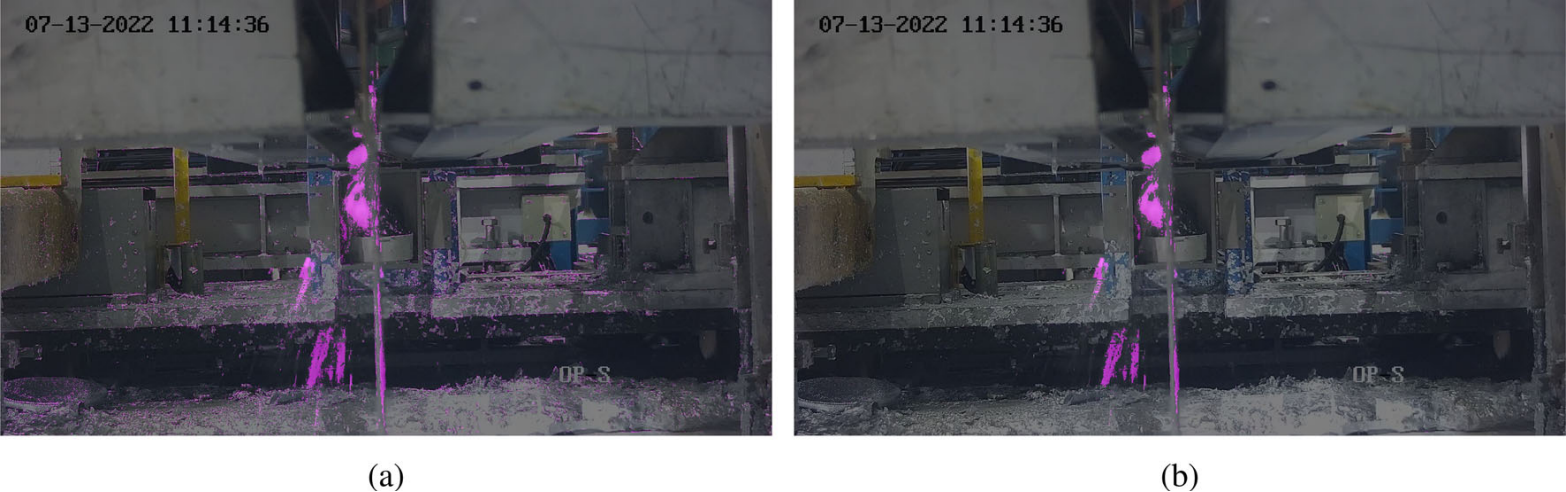
Effects of contour erosion on CNT background segmentation

As previously mentioned, trial-and-error experimentation was conducted to optimise the CNT algorithm parameters for this application and solve the issue with knife movement. The parameters targeted were minimum pixel stability and maximum pixel stability. Minimum pixel stability is the minimum number of frames with constant pixel colour to be considered stable for segmentation, whereas maximum pixel stability is the maximum allowed historical credit for a pixel (OpenCV, 2023) Based on the definition of minimum pixel stability, the value needed to be very low (0–3) to ensure the algorithm was sensitive to pixels changing across every single frame since the splatter often changed significantly between frames. Based on the definition of maximum pixel stability, the value needed to be low enough to only detect small changes in the video and ignore occasional changes such as air knife movement, whilst being high enough above the minimum value to allow for more slowly developing splatter. The final values for minimum and maximum pixel stability were set at one and ten respectively and the before and after results of this optimisation are shown in Figs. 8 and  9 respectively, which use the same frames for comparison. The results show optimising the CNT parameters was successful in removing the air knife segmentation since the minimum pixel stability was low enough to detect small changes in splatter from frame to frame, whilst the maximum pixel stability was low enough to ensure the background pixels are not calculated based on many previous frames so air knife movement was accounted for. Also, inference time was reduced marginally from 0.0505s to 0.05s.

Finally, erosion was applied with a (2,2) filter in order to remove small background noise due to heatwaves and camera shaking which makes the mask much clearer. The results are shown in Fig. 10 where (a) shows no erosion applied and (b) is after erosion has been applied.

### Splatter severity measurement

Once the model was capable of extracting the splatter the next step was to properly quantify the severity of it. This was fundamentally broken down into two factors: splatter amount (number of segmented pixels in the splatter region) and splatter width (how widespread the segmented pixels in the splatter region were). The splatter region was the area in the video specified as being expected to contain splatter, and was applied to minimise the possibility of noise that occurs in an area where splatter never exists affecting results. The region was defined based on the results of object detection which are presented in the next subsection. Also, a contour threshold of 75 pixels was used to ignore tiny amounts of splatter that spread far wider than the majority of the remaining splatter signature meaning the severity rating would be unrepresentative of what is observed.

**Fig. 11:**
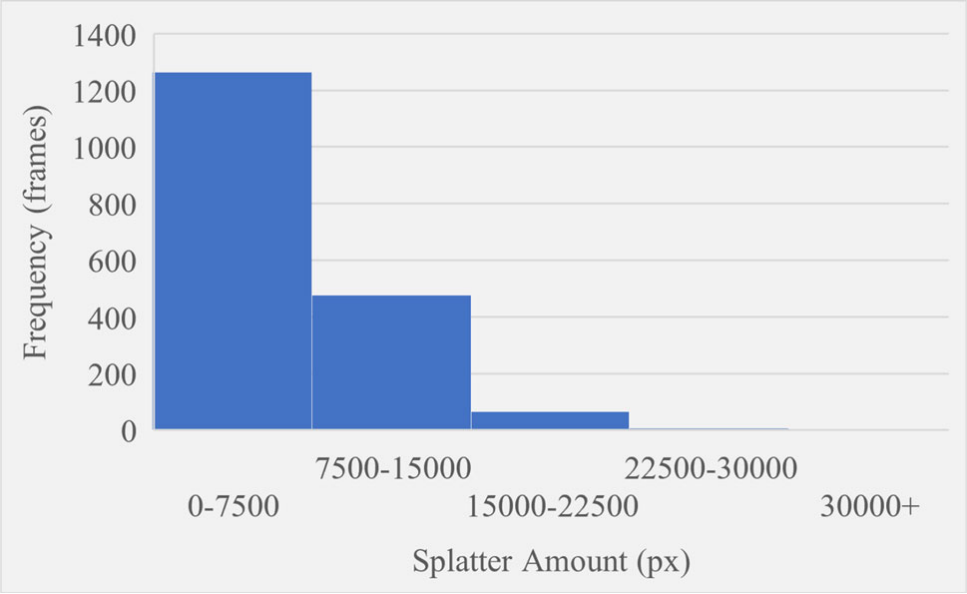
Histogram showing how frequent different splatter amount values occurred

**Fig. 12:**
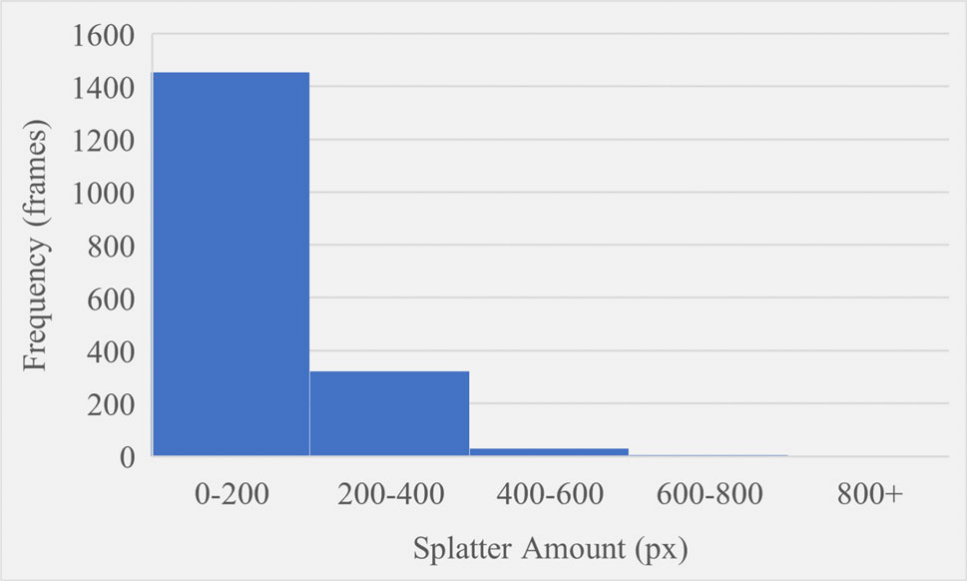
Histogram showing how frequent different splatter width values occurred

The splatter amount and width were recorded for every frame of Video 1 and plotted as the histograms shown in Figures 11 and 12 respectively. Splatter amount ranged from 0 to 42655 whilst splatter width ranged from 0 to 847. Both histograms have been intentionally divided up into five different ranges that define the boundaries for the severity levels of splatter amount and splatter width. For both variables, the first severity level is what the majority of frames are measured as since this is the baseline state of the process. The second bin contains significantly less frames than the first however it contains significantly more than every subsequent bin, and this represents a severity of one. The third bin is the last that has a high enough frequency to be visible on the chart and this represents a severity of two. The fourth bin is invisible and contains only seven and five frames for splatter amount and splatter width respectively. The final bin defines a severity level of four and has no upper limit to account for any extreme cases. For the final bin, six and four frames were recorded for amount and width respectively. Figures 11 and 12 show the trend of higher severity levels being less likely to occur, which is desirable as this is generally the case in real-world scenarios. Finally, the rating system previously shown in Fig. 4 within “Methodology" section was used to give the final splatter severity rating for each frame. Examples of severity ratings from the final model are shown in the next section.

### Object detection

#### Model training

For training, the model was evaluated based on bounding box, object and classification losses for training and also validation at the end of each epoch. Precision, recall and mean average precision were also recorded for validation results. For testing, the same metrics were used for comparison.

**Fig. 13:**
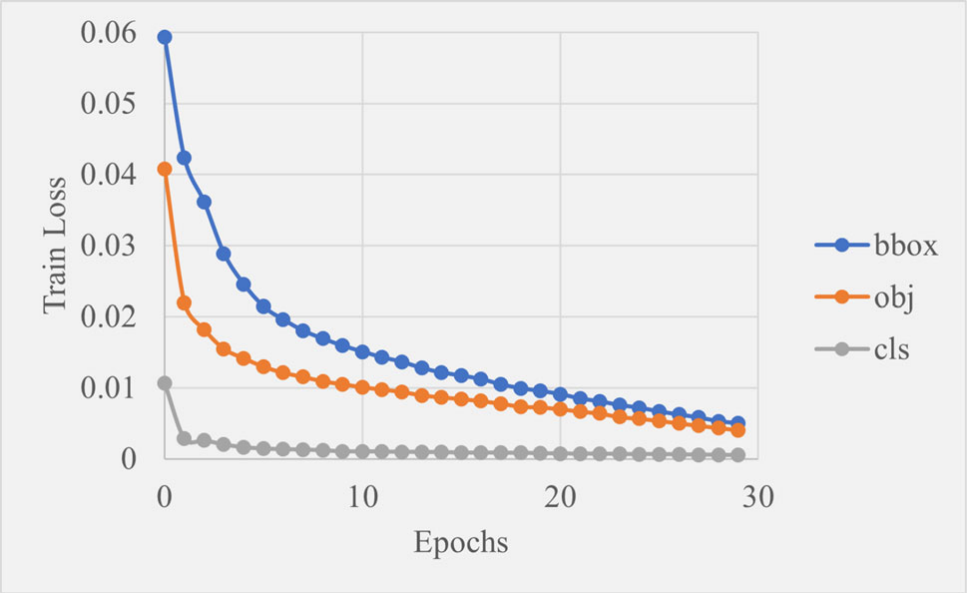
Graph showing how training loss changed over 30 epochs of training

Figure 13 shows the training losses for bounding boxes, objectness And classification decreased gradually over the 30 epochs with the same trend. This is a typical shape for a loss versus epoch graph during successful training as it shows the model gradually improved its ability to predict bounding box locations, object presence and class labels through learning the features existing within the training data.

**Fig. 14:**
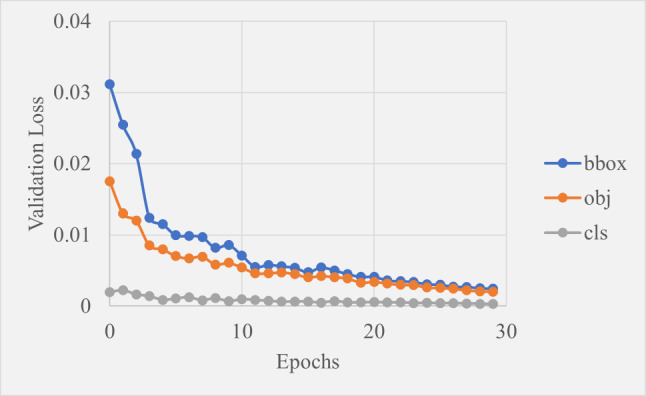
Graph showing how validation loss changed over 30 epochs of training

Figure 14 shows the validation losses which followed the same pattern as the training losses with slightly lower values overall. This is atypical since validation losses are normally slightly higher, however it is unproblematic. It could be due to the data distribution between training, validation and testing sets that meant the validation set is easier to predict on than the training set. The decreasing trend shows the model successfully learned from the training set and applied that knowledge to the validation set. There is some noticeable jaggedness of the validation lines compared to the smooth training lines, which is expected due to the model adapting to unseen data.

Table 3 shows the precision, recall,  and  were all almost perfect which was expected due to the large training set and minimal movement or environmental changes, excluding the splatter. This aligns with the loss graphs which as discussed, both indicate successful training.

**Table 3:** Validation Results

Precision	Recall	mAP_COCO_
0.99988	1	0.99414

**Fig. 15:**
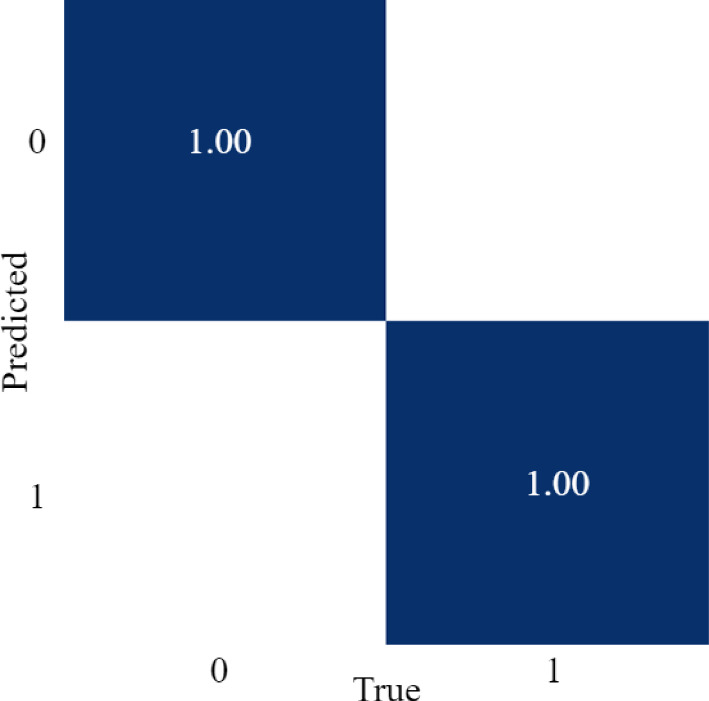
Confusion matrix showing testing results

**Fig. 16:**
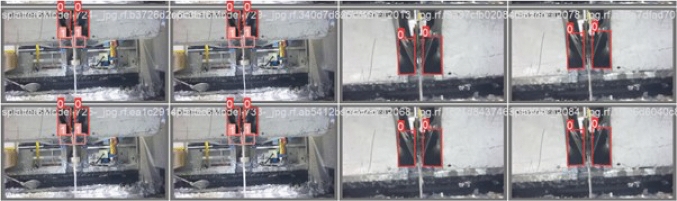
Visualisation of testing results (one class shown)

#### Model testing

**Fig. 17:**
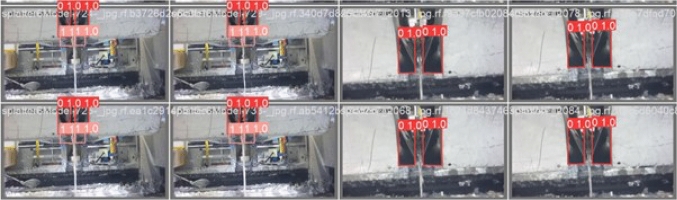
Visualisation of testing results (two classes shown)

Figure 15 shows the confusion matrix which shows true positives and false positives for both classes. All instances were predicted correctly. Figures 16 and  17 show labelled frames and corresponding predictions.

The testing results are shown in Table 4. In comparison to the validation results, model performance was virtually identical, which is a good indicator of it working well during deployment. The model was trained on all camera positions that it was tested on for a minimum of 750 frames and there was not much variation between positions. New positions will be experienced during deployment, however there is high confidence that the model will still generalise well due to learning from extremities and interpolating for unfamiliar perspectives. If not, the model will be trained on a wider range of positions until it performance is satisfactory.

**Fig. 18:**
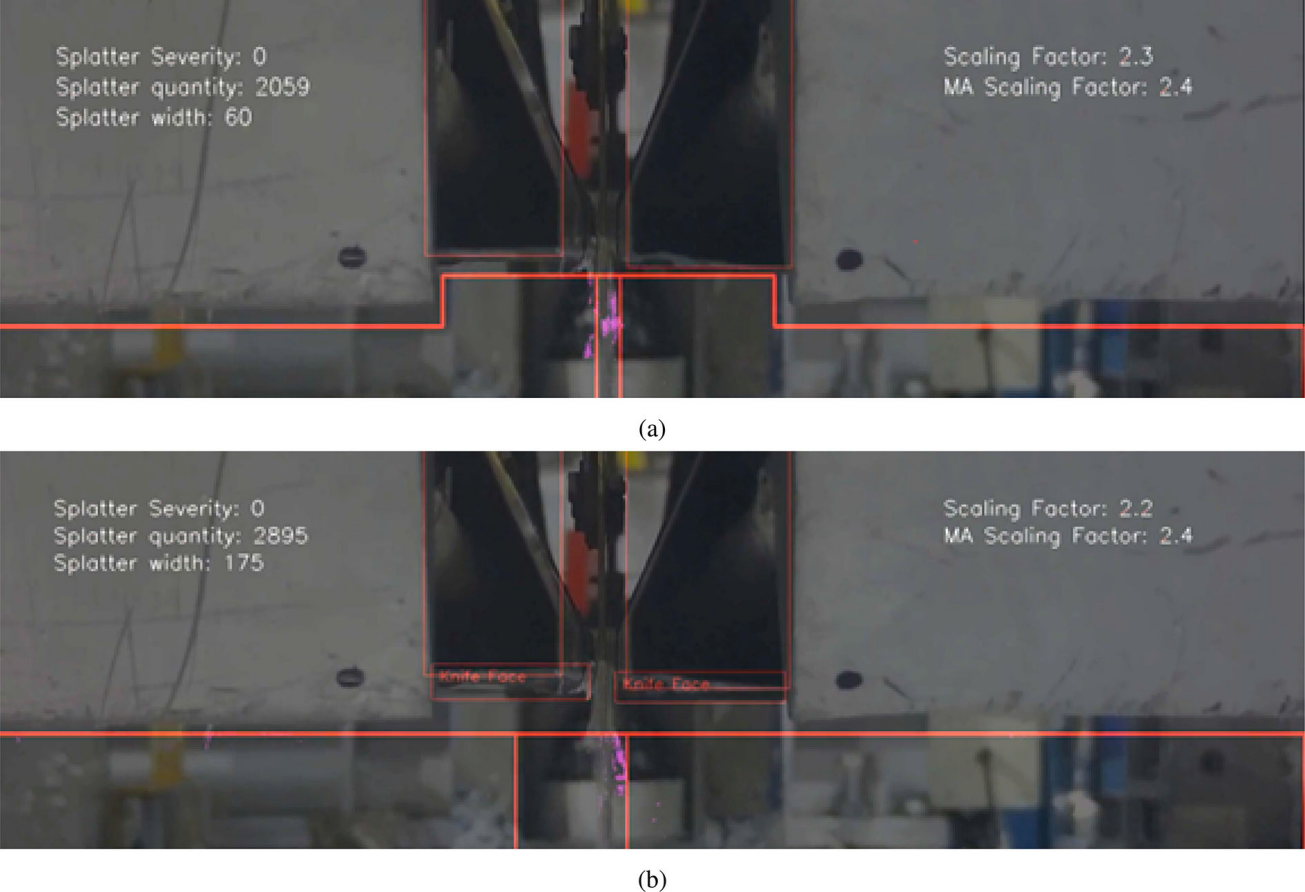
Demonstration of splatter region line changing depending on how the knives are positioned

#### Splatter region

Once object detection was successful, the next step was to properly define the splatter region. Figure 18a shows how the splatter line (which was originally always straight) has been optimised to follow the shape of the knives. The bounding boxes allowed enabled line optimisation and also meant the model could deal with knife movement. When the underside faces of the knives appear, the line straightens out as shown in Fig. 18b, to avoid the walls entering the splatter region. Also notable, is the two vertical red lines on either side of the segmentation mask which shows exactly where splatter is being measured and the distance between the two lines is the splatter width.

#### Scaling factor

Bounding boxes were beneficial for defining the splatter region, but also for improving model robustness in terms of variations in camera position. Operators at the coating site regularly move the cameras and so the model was better adapted for use when having this capability. However, to ensure the most reliable readings from the model, it is advised that the camera is kept as similar to an optimal reference position which is that used to produce Video 1. As explained in “Methodology" section, different camera positions used for developing the model in order to account for this.

Robustness to camera position was further achieved by using a scaling factor. The area in pixels of the combined bounding boxes for the left and right knives of the optimal reference position was used as a reference area, and then depending on the distance between the camera and the knives, the bounding boxes increased or decreased in size and therefore increased or decreased the scaling factor which can be seen in Eq. 6 of Sect. 3.

**Table 4:** Testing Results

Precision	Recall	mAP_COCO_
0.99989	1	0.99449

**Fig. 19:**
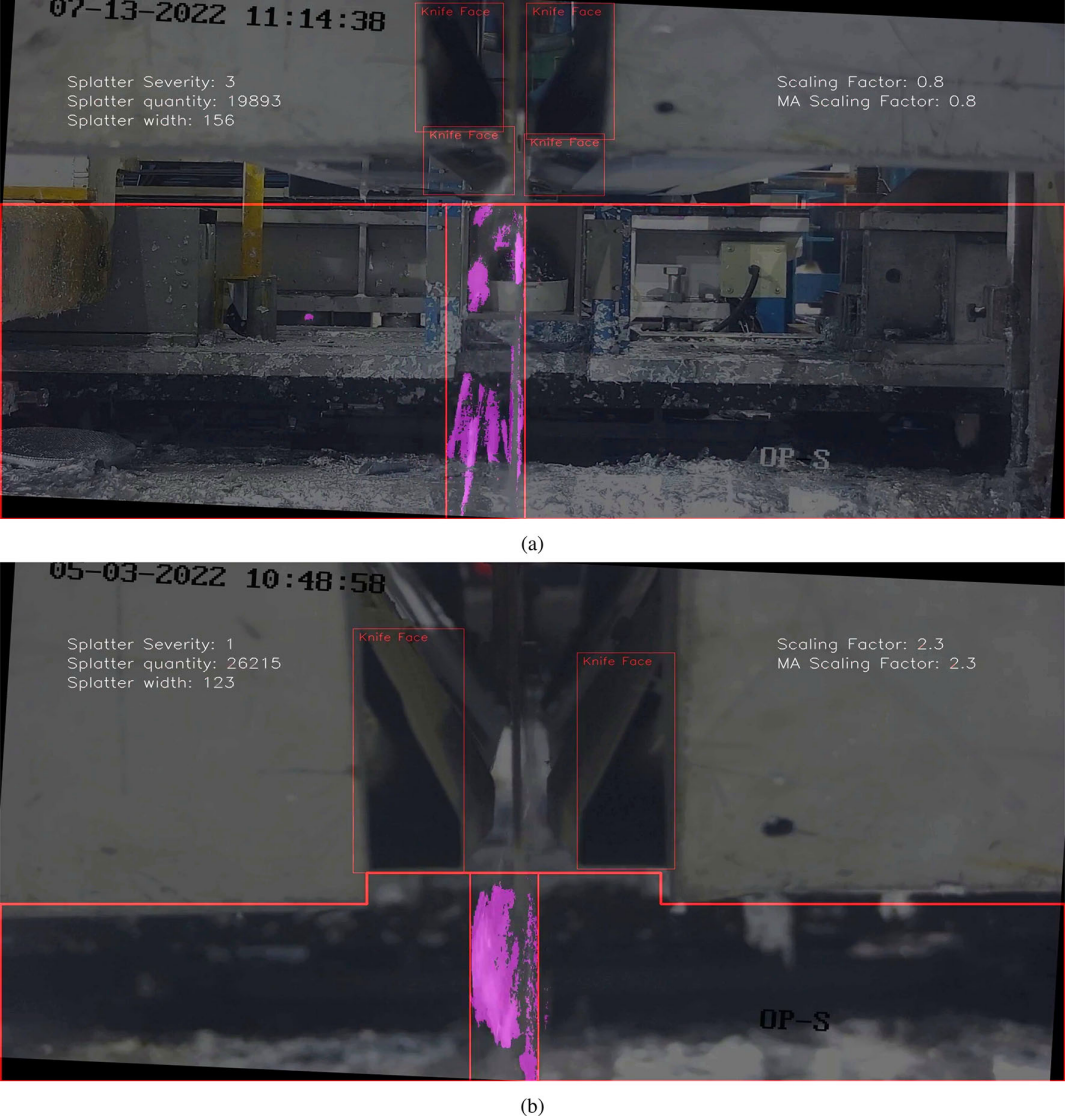
Demonstration of splatter measurement boundaries adapting to changes in camera position

The scaling factor was used as a multiplier on the splatter amount and splatter width severity level boundaries to ensure changes in distance between the camera and knives did not cause splatter severity to be over or under-exaggerated. Figure 19a and b show results on two different camera positions and whilst (b) shows a similar splatter amount and splatter width, the severity rating is still low since the camera is closer to the knives and therefore only giving the appearance that there is more severe splatter.

After implementing the scaling factor, it was decided that a 25000-point moving average would be used. The reason for this value was that at 25fps, the MA scaling factor will be updated every 1000 seconds which is just under 17 minutes. It is expected that the operators will move the camera no more often than every few hours so this provides a safety factor.

### Final evaluation

After the entire prototype was developed it was necessary to test it on the seven 60-second videos that were set aside initially to ensure the model was as close to production-ready as possible.

For Video 1, the camera-knife distance was roughly at the reference point for the scaling factor and contained the entire range of severity levels. The model performed well throughout and there were only a handful of frames from the whole video that contained clear inaccuracies due to noise, and these could easily be detected as anomalies and removed or ignored when interpreting the data for process optimisation.

For Video 2, the camera-knife distance was over double the reference point for the scaling factor and contained mostly low severity levels but also exhibited higher severity at times. The model performed well with minimal inaccuracies due to noise.

For Video 3, the camera-knife distance was about half the reference point for the scaling factor and splatter severity was mostly low. Inaccuracies were minimal.

For Video 4, the camera-knife distance was normal and the camera was positioned so that the zinc pool could be seen clearly. The flowing zinc was detected by the CNT algorithm due to constant movement, which made the model massively inaccurate. A way of enabling the CNT algorithm to segment splatter accurately but ignore the flowing zinc pool is yet to be proposed, however one solution and the one taken for this deployment is to accept the limitation and ensure the camera is always positioned so that the pool cannot be seen, which is acceptable since it normally cannot be seen.

For Video 5, the camera-knife distance was about half and only low severity levels were exhibited. There was some light reflecting off an area to the right of the air knives which could not be seen in other videos. Similarly to the zinc pool also discussed, this is currently an accepted limitation handled by ensuring the camera is positioned to eliminate the noise source from view. it is possible that the light could be detected or segmented and then eliminated, but this would take tedious and potentially difficult model development which can be done just by camera position adjustment. The video also showed some horizontal knife movement to which the object detection element of the model handled well, and the splatter region was adjusted accordingly.

For Video 6, camera-knife distance was normal and a range of severity levels were seen. Similarly to Video 5, the camera was angled to the right slightly which exposed reflecting light that often occurs just outside the optimal field of view. Other than the noise caused by this, the model performed well.

For Video 7, camera-knife distance was over double and only low severity levels were seen. This video contained vertical air knife movement from the bottom to the top of the camera”s view. The model competently adjusted the splatter region as the knives moved upwards and the transition between only front knife faces being visible to front and underside faces was fairly smooth.

A final important finding found during the final production testing was that despite having near perfect performance during training, validation and testing, the model struggled with distinguishing between classifying knife front faces and knife undersides. In some frames of some videos, whilst the bounding box predictions remained near perfect, the model switched classes for the boxes incorrectly. The reason for the discrepancy between the model development and final testing results is probably due to the size of the validation and/or testing sets being too small in comparison to the 15000-frame production set, or just insufficiently representative of it. This is not an issue for deployment, since the box predictions are reliable enough to be able to inform the model that the underside faces are visible if there are two boxes predicted under the main (front face) boxes. However, this is definitely an area for improvement, especially if the model is developed further or adapted to other applications.

### Expert validation

Table 5 shows the MAE between model predictions and the first operator (PO1), the model and the second operator (PO2), and between PO1 and PO2. From Table 5 it can be seen that firstly, the model”s maximum MAE across both operators is 0.95. This implies it is unusual for the model to be predicting splatter severity as more than one level away from what an expert observer would say, which is promising. Secondly, the MAE between the two operators was higher than the MAE between the model and PO2. This suggests that either the element of subjectivity has caused a wide variation in results leaving a high chance of high variability between operators, or the model is more accurate than one or more of the operators. The significance of the differences between MAE values could be further established by extensive testing to learn more about the degree of random variability between operators and whether certain datasets, environmental conditions or the experience level of selected operators result in divergence from model predictions.

**Table 5:** Mean average error values between different validation test results

Model-O1 MAE	Model-O2 MAE	O1-O2 MAE
0.95	0.60	0.95

**Fig. 20:**
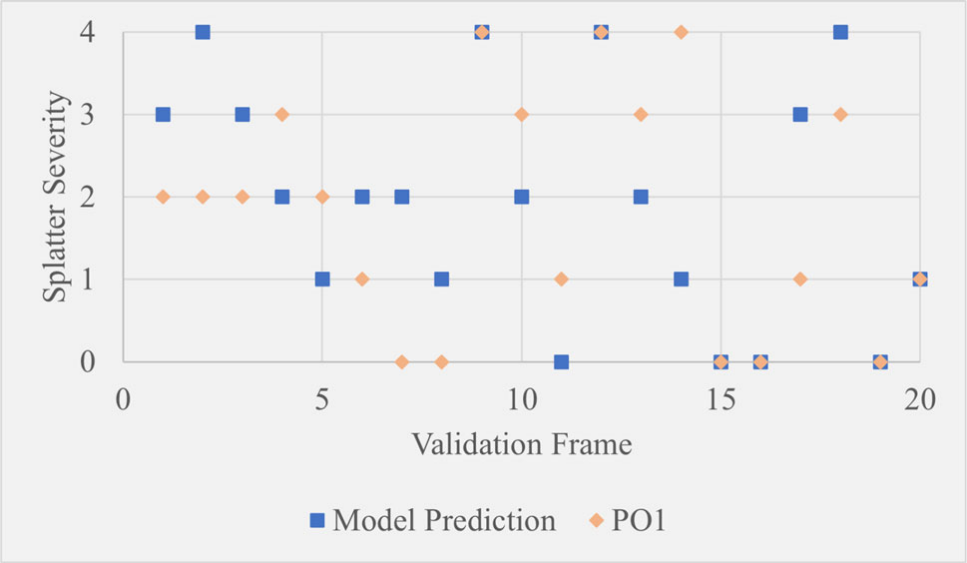
Scatter plot showing model predictions compared to PO1 over 20 hand-selected validation frames

**Fig. 21:**
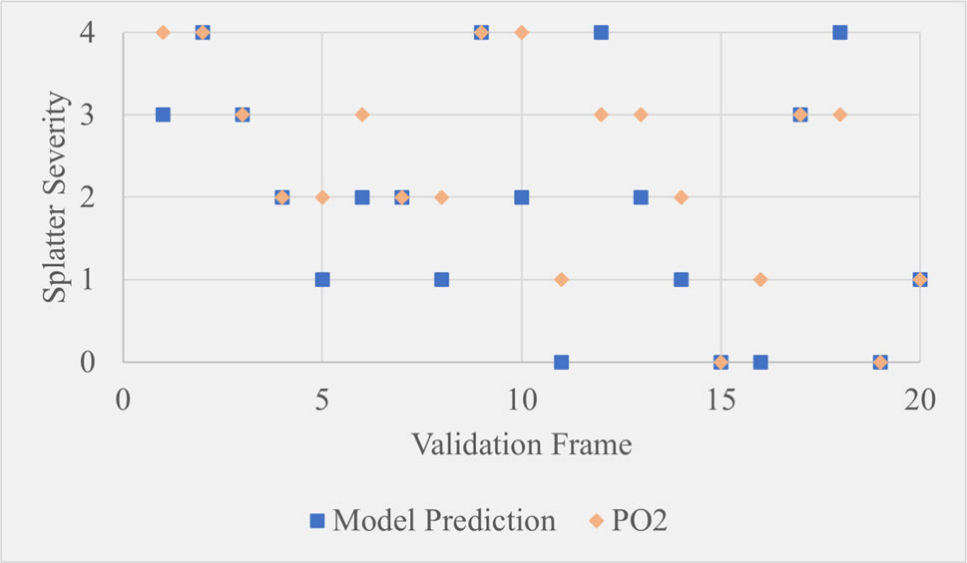
Scatter plot showing model predictions compared to PO2 over 20 hand-selected validation frames

**Fig. 22:**
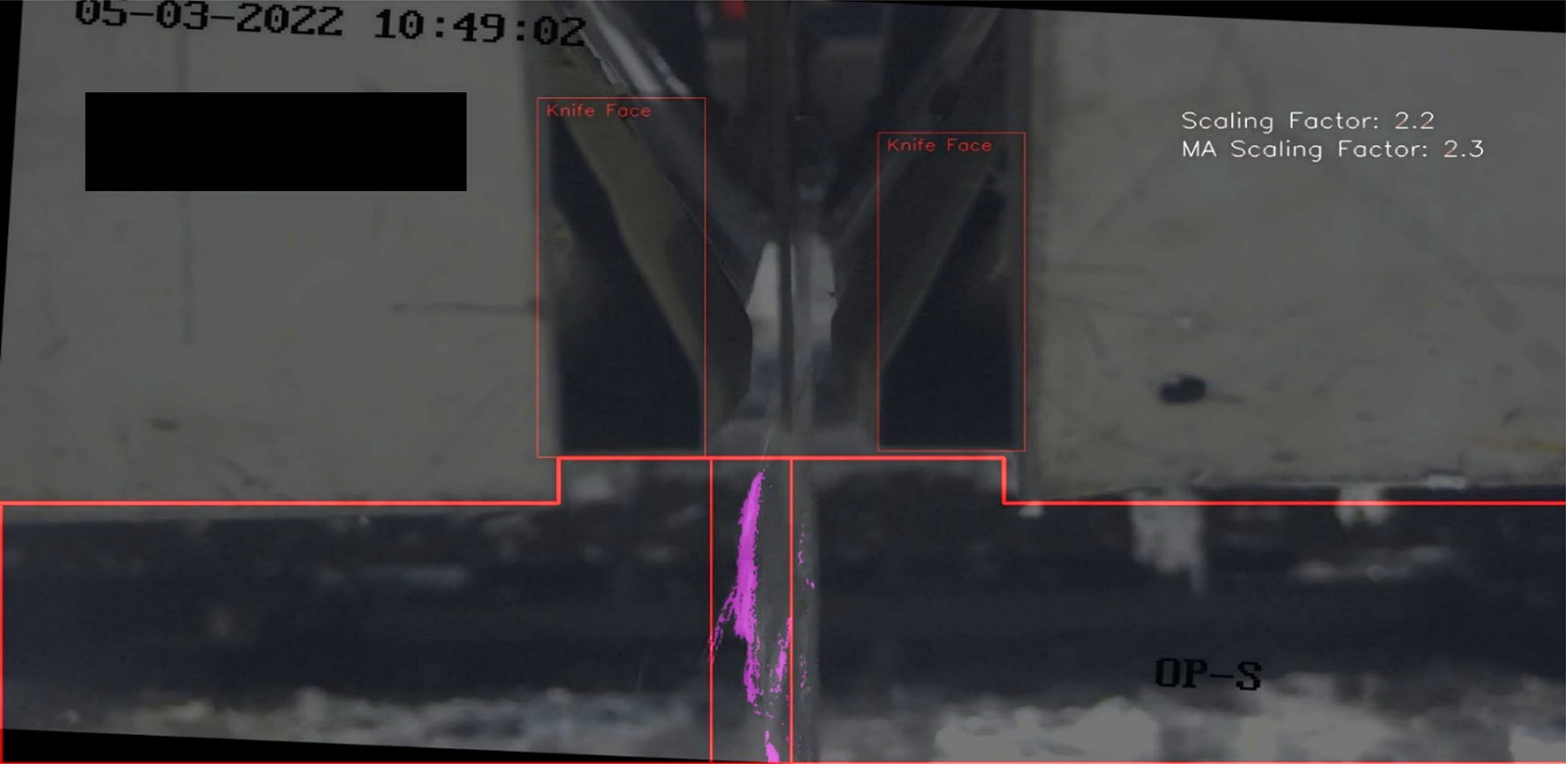
Frame 11 in the expert validation sample set where a discrepancy was found between operators and the model

The scatter plots in Figures 20 and  21 show a frame-by-frame comparison between the model and operators 1 and 2 respectively. While the results show that the model”s predictions did not consistently lean towards PO1 or PO2, they were noticeably closer to PO2. The fact that the model generally sits between the opinions of both operators suggests it could be useful as a reference point when operators disagree. The subtle leaning to PO2 suggests the assessment criteria during model development was more similar to PO2 than PO1. Additionally, the model predicted outside of both operator judgements by one severity level six times. These were on frames 5, 10, 11, 13, 14 and 18. All of these except frame 18 had a scaling factor of approximately 2.3 (camera close to the knives), suggesting that the camera position at the initial stages of model development resulted in accurate predictions, whereas scaling factor multipliers that accounted for camera position were not optimal. This is further supported by the fact that of the 20 sample frames, seven of them had this scaling factor.

Figure 22 shows frame 11 within the expert validation set. The model predicted a severity of zero, whereas PO1 and PO2 judged the severity as one. As shown in the image, the CNT algorithm did not detect a large amount of the zinc, however there was a minute amount of splatter visible. There was bias built into the model during development as it could have been decided that any amount of splatter segmentation raises the severity from zero to one, however this was not the approach taken which evidently resulted in the discrepancy. Also, Fig. 22 shows that the model may struggle to differentiate between very small amounts of splatter and no splatter, which is an area for future improvement but is not critical to the model application.

**Fig. 23:**
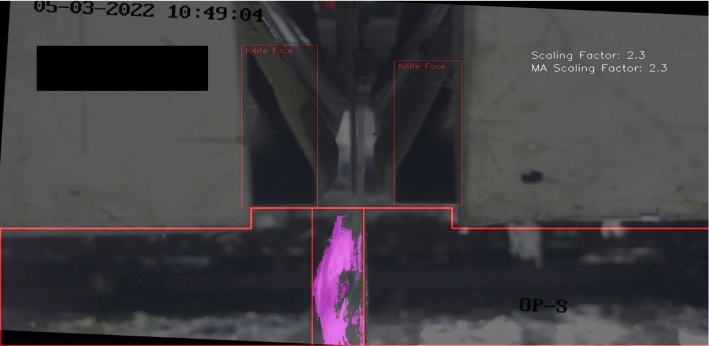
Frame 14 in the expert validation sample set where a discrepancy was found between operators and the model

Figure 23 shows frame 14 within the expert validation set. The model predicted a severity of one, whereas PO1 and PO2 judged it as four and two respectively. Of all six discrepancies this was the most significant since the difference between PO2 and the model was three severity levels which is large. The results show that firstly, the discrepancy between the PO1 and the model was one severity level, which as previously mentioned, was likely due to the scaling factor multiplier being slightly misaligned with operator judgment. Secondly, the difference between PO1 and PO2 was significant and by looking at Fig. 23, as well as considering the model did not disagree with any operator judgement in any other frame by more than two severity levels, the value given by PO1 was misjudged.

**Fig. 24:**
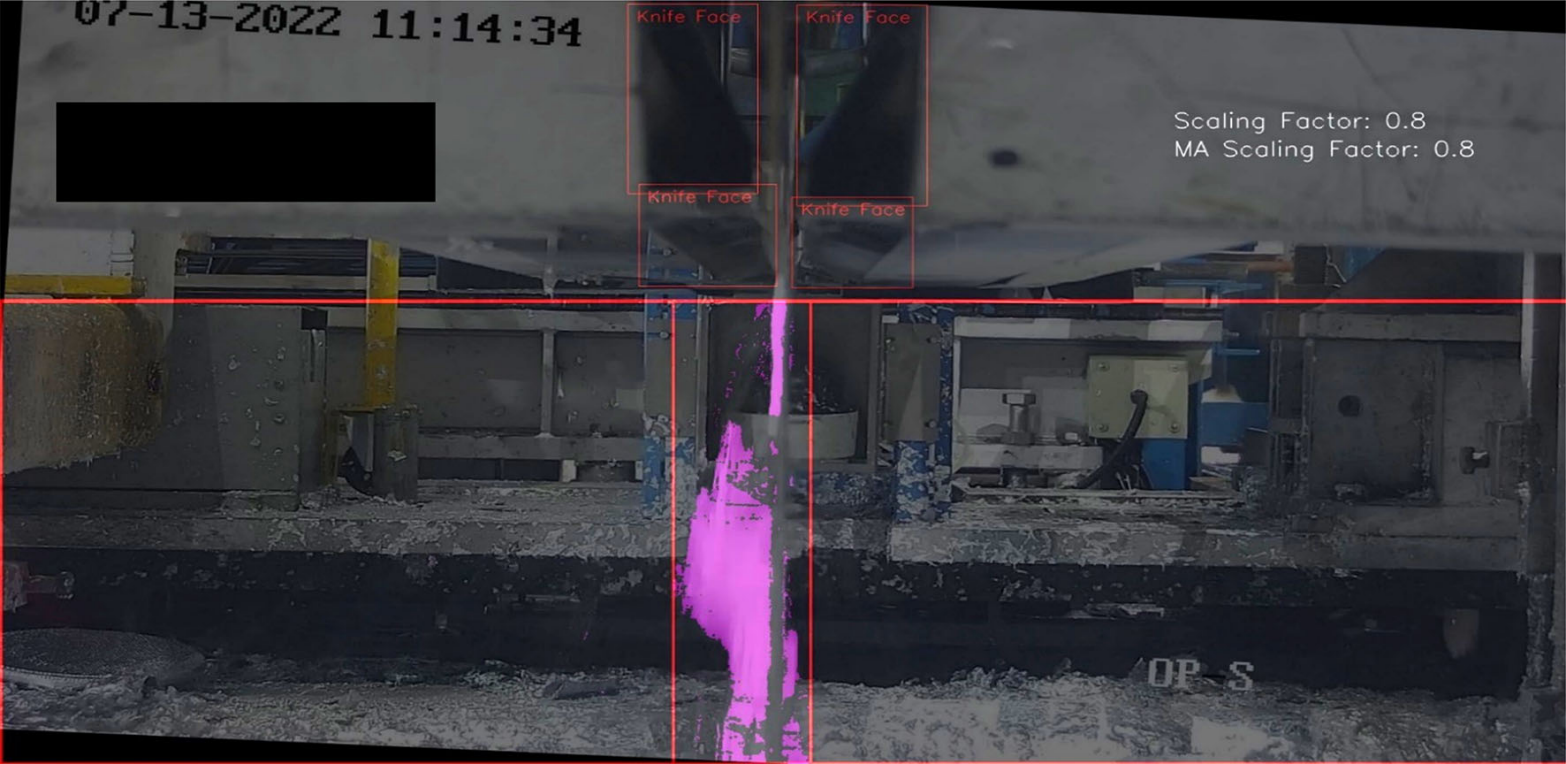
Frame 18 in the expert validation sample set where a discrepancy was found between operators and the model

Figure 24 shows frame 18 within the expert validation set. This was the only clear discrepancy of the whole expert validation set that was not based on a scaling factor of 2.3. In this case, the model predicted a severity of four whereas both operators judged it as three. The discrepancy here appears to be due to built-in bias when choosing severity level boundaries. The model has been built to consider this amount of splatter as the highest severity level, whereas the operators have considered it the second highest severity level, which is certainly more accurate by observing Fig. 24. This analysis contributes to model refinement.

**Fig. 25:**
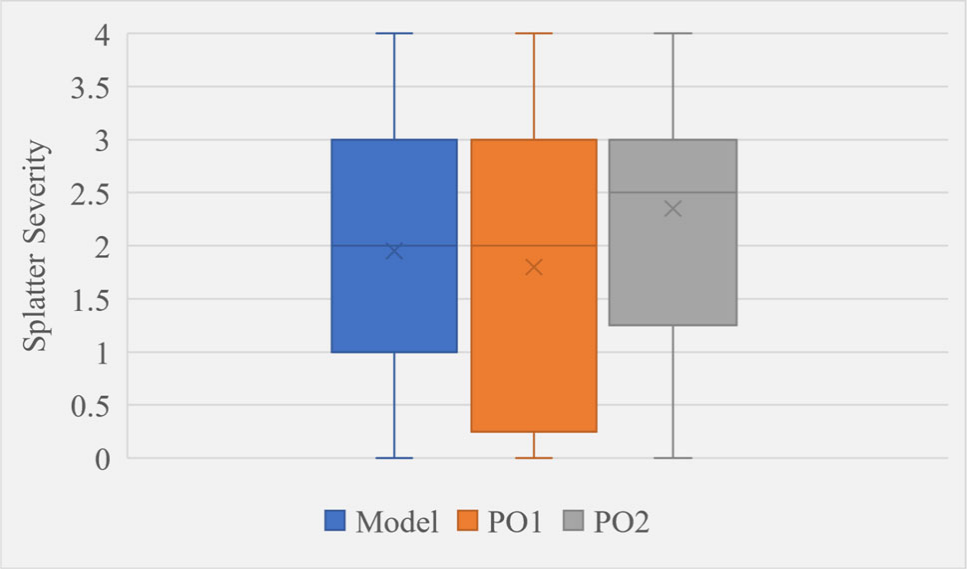
Box and whisker plot showing model prediction distribution compared to two different operators over 20 hand-selected validation frames

The box and whisker plot in Fig. 25 further explores model-operator relationships. The boxes represent the interquartile ranges of each results set, whilst the whiskers represent the range of potential values which was always zero to four. The boxes show that the distribution of the model”s severity predictions was between both operator”s distributions. The interquartile range of the model and PO2 were most similar, whilst the medians of the model and PO1 were most similar. These results suggest the model is effectively capturing operator”s observational tendencies and indicate the potential for the model to serve as a robust tool for standardising severity measurements in application.

**Fig. 26:**
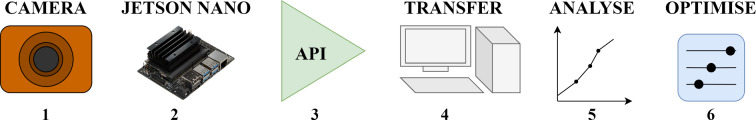
Representation of the intended workflow for deployment

In conclusion, the model has demonstrated promising accuracy in splatter severity prediction, however there is noticeable variability between operators which possibly exposes an underlying complexity of manual evaluations. The results suggest that the model is suitable for assisting experts and could serve as a standardisation tool for splatter severity assessment. To further refine the model and ensure it not just replicates but surpasses expert precision, a more in-depth study on expert judgment would be critical.

## Industrial application

The model will be deployed near to and facing the air knives which themselves are above the molten zinc bath where coating occurs. The model will be deployed using a NVIDIA Jetson Nano (NVIDIA, 2023), which is a single-board computer that has been designed specifically for edge AI and deep learning applications. It is comprised of a quad-core CPU, a NVIDIA GPU with 128 NVIDIA CUDA cores as well as internet connectivity, camera connectivity and more. Collectively, these components allow running of computationally demanding tasks such as recording live video footage and performing real-time CV model inferences on it (NVIDIA, 2023). The camera input is resized to speed up processing to ensure real-time which consequently means elements such as severity level boundaries for amount and width are scaled accordingly. Using an NVIDIA RTX 2070 Super the model ran at 8fps with the raw frame size (1920px by 1080px) and 15fps with the frame size at 20% and similar effects are expected with the Jetson. Whilst the 2070 Super is built for high performance computing and gaming, the Jetson is designed for more lightweight applications in resource-constrained environments. Therefore, the Jetson is expected to perform at a lower frame rate of approximately 5fps, which means the overall system will be capable of measuring one in five frames in comparison to the original footage.

Figure 26 shows the workflow for the device. The workflow begins with the API (application programming interface) which wraps around the model and allows operators to interface with the model without having to understand the model code. The API takes the operator input and communicates it to the device which takes in raw data from the camera.

The model will run as normal using the camera as input and will only save the timestamp and splatter severity value for each frame rather than the processed video output, splatter amount, splatter width and scaling factor. This will ensure the minimal data necessary is stored for storage efficiency. Each frame will save two values; the timestamp which will be in Unix timestamp format (32-bit integer which is 4 bytes) and the splatter severity which will be in integer format (1 byte), making each frame adding 5 bytes to storage. At full real-time speed there are 25 frames per second, meaning there are 125 bytes stored per second, which means in order to completely fill the 16GB storage on the Jetson Nano, the model would need to run for over 30000 hours without deleting measurements. Regardless of this, the main storage facility will be an external computer that receives the output over a TCP/IP connection which the Jetson connects to via an Edimax N150 Wi-Fi Nano USB adapter (Edimax, 2022), which not only prevents data storage issues but also means the full output video could be saved for particular experiments, or as a troubleshooting mode

The severity values will be analysed by operators to look for hidden trends and relationships between process parameter values and splatter severity. These trends and relationships will then be used to optimise the process parameter values during operation to minimise the splatter severity at high strip speeds. The workflow of the model is presented in Fig. 26.

Whilst the concept of objectively quantifying zinc splatter severity using technology is entirely novel and therefore has no alternative technologies, comparisons can still be drawn between the approach presented in this paper and current practice. The current approach is for operators to judge by eye and give an entirely subjective opinion on how severe the splatter is. This can be affected by a wide variety of factors such as the operator’s position relative to the air knives, their experience, and even their mood on that particular day. Oppositely, the approach presented in this paper is entirely objective, measures splatter severity the same way every time and gives a definitive number on a scale of zero to four, making it more reliable and informative. Also, the consistent measurement of the proposed approach means it can be used to standardise measurements which is beneficial for optimising the galvanising process over long time periods. Additionally, splatter monitoring is performed automatically which after deployment, reduces resource requirements. Previous discussion emphasises the benefits of using this approach in comparison to the current practice, however, it is also important to consider some of the limitations of this approach. One limitation is the initial setup and training of the model which is time-consuming and resource-intensive. This could pose a challenge for small and medium-sized enterprises (SMEs) with limited computational resources and expertise. Meanwhile the current practice relies purely on operators that are already present at the site. Furthermore, to adapt the model to different manufacturing environments would currently require retraining on new data which poses challenges with regards to how quickly the model can be deployed effectively. Therefore, the flexibility of using human judgement is advantageous over the proposed approach. Despite these limitations, the advantages of an automated, objective system outweigh the relatively low resource requirements of the current practice.

The potential impact of this model can be broken down into technological, environmental, economic and social benefits. Technologically, advantages brought by the device are vast. Firstly, a previously unquantifiable variable, splatter severity, is now measurable. This brings new possibilities for root-cause analysis, preventive maintenance, predictive maintenance and process optimisation. Finding the cause of the splatter will be easier since it will be measured as changes are made to the process. Preventive maintenance in the form of cleaning will be easier to manage since the accumulative recorded splatter over a given period of time will indicate how much has collected on the surrounding equipment, whilst predicting when maintenance is due will be easier since the amount of splatter accumulated will be approximately known. Also, the recorded data can be post-processed to give various plots and analyses that show previously undiscovered trends in the system related to splatter. Trends will suggest process changes that reduce splatter at high strip speeds, leading to less equipment downtime and increased productivity. Finally, the model concept is novel and uses cutting edge CV algorithms in combination to monitor a previously unrecorded process variable in real-time. It therefore provides an initiation point for future research and development related to CV applications for manufacturing, particularly those which involve liquid that changes morphology at a fast pace. An example of a potential feature that could be added to this model includes measurement of the air knife distance to easily look for relationships between distance and splatter severity. This could be developed further to fine tune air knife distance autonomously in real-time to minimise splatter severity. Some examples of potential applications in other contexts firstly include automated spray assessment for coating aircraft components. Overspray leads to material wastage and increased costs, while underspray reduces performance, meaning automating this could improve quality control and sustainability of the process. Secondly, leaking or spraying detection along food and drinks manufacturing lines could make production processes more adaptable in real-time, therefore improving production efficiency and minimising waste. Thirdly, lubrication spray assessment for real-time feedback on coverage, volume and consistency of the lubricant applied to machinery to ensure optimal application which prevents excessive wear and decreases downtime, therefore maintaining high production standards. Not only is this work beneficial for the manufacturing sector, but also for industrial CV. For example, this work demonstrates the benefits of using hybrid models that capitalise on the advantages of both deep learning and more traditional CV techniques to make models more adaptable, therefore encouraging further research and development in this area. Also, this work emphasises the potential for integrating CV models with IoT devices such as the Jetson Nano for in-situ monitoring of industrial processes. Furthermore, the importance of real-time data analysis along manufacturing lines for instant feedback is highlighted, which contributes to faster decision making and improved efficiency.

By informatively driving process optimisation, the splatter measurement device is beneficial for the environment since less defects occur, meaning less energy and material is used to output the same amount of galvanised steel. Consequentially, economic benefits are that the steelmakers will no longer have to pay as much for energy or material for a given production rate.

Regarding social impact, the workers will be required to clean less zinc that has splattered off the strip and onto the floor, air knives and electromagnetic stabilisation system which eases workload. It also improves health and safety since there is less time where workers are cleaning close-up to the equipment which is hazardous. Furthermore, the automatic real-time measurement of splatter severity using this model is a far superior measurement approach compared to the traditional method of observing by eye. This could not only reduce workload but it will definitely improve the confidence and awareness of workers making decisions based on splatter severity which makes their job easier.

## Conclusions

This paper has illustrated the development of a model that can be used to monitor, and therefore quantify, the severity of molten zinc splatter occurring along the galvanising line during bath immersion due to air knife application at high strip speeds. Once a prototype was developed, industrial application and model deployment were considered and a production-ready device setup has been proposed. The best background subtraction algorithm for this application was the Counting algorithm with minimum and maximum pixel stabilities of one and ten respectively, whilst YOLOv5 was suitable for the application with some notable room for improvement on the multi-class element of the problem. Despite this, the model was built to a deployment-ready level of development and a plan for the remaining implementation stage has been proposed. The recorded precision, recall and mAP_COCO_ were 1, 1 and 0.99 respectively on the test set.

This research significantly contributes to the fields of both manufacturing and CV as it binds CV techniques such as object detection, background subtraction and image processing, with manufacturing elements of root-cause analysis, process optimisation, and maintenance planning, to not only develop real, applicable device that solves a real-world problem with certified value, but also strengthens the research and development space where it can be used as a starting point to be built upon and used to inspire similar applications that could revolutionise multiple industries.

Potential future developments on the model presented in this paper were mentioned briefly in “Industrial application" section, and so a few applications will be mentioned. Firstly, the techniques used here would be ideal for developing an automatic pipe leak detection system where may be useful to automatically indicate the size, flow behaviour and morphology of the leak using background subtraction. Similarly, a safety system that detects fires could be built using the object detection to recognise fire whilst using the background subtraction to reduce false positives caused by transient changes. These are just two possible applications that underline the versatility of the approach proposed in this paper.

Some limitations of this approach were mentioned in the industrial application section and should be addressed in future works. Despite the effectiveness of the training process, it requires investment of resources which may not be viable for SMEs. These mainly involve data and training requirements, and therefore future work should explore methods for enhancing the scalability and adaptability of the approach. One promising approach is the use of transfer learning which significantly reduce data requirements and training time by adapting pre-trained models to similar situations with a small amount of retraining.

## Data Availability

The data that support the findings of this study are available from the corresponding author upon reasonable request.

## Code availability

Code used in this study may be available from the corresponding author upon reasonable request.
